# *TMEM189/PEDS1* deficiency aggravates MASH progression by promoting FASN-mediated macrophage activation

**DOI:** 10.1038/s41419-026-09145-7

**Published:** 2026-08-13

**Authors:** Pengli Guo, Jinqiu Feng, Yaxin Lou, Tao Li, Shufang Ye, Yongjun Zhang, Chunlei Wang, Ping Lv, Zongming Zhang, Yingyu Chen

**Affiliations:** 1 Department of Immunology, School of Basic Medical Sciences Peking University; NHC Key Laboratory of Medical Immunology, Peking University, Beijing, China; 2 Department of General Surgery, Beijing Electric Power Hospital, State Grid Corporation of China; Clinical Medical Research Center for Hepatobiliary Diseases in General Surgery, China General Technology Group, Beijing, China; 3 Medical and Healthy Analytical Center, Peking University, Beijing, China; 4 Center for Human Disease Genomics, Peking University, Beijing, China

**Keywords:** Chronic inflammation, Metabolic disorders

## Abstract

Transmembrane protein 189 (TMEM189), also known as plasmanylethanolamine desaturase 1 (PEDS1), is involved in plasmalogen biosynthesis, phospholipid metabolism, ferroptosis, and autophagy. However, whether TMEM189/PEDS1 regulates macrophages and disease-related inflammatory responses remains unclear. Here, we for the first time investigated the contribution of TMEM189/PEDS1 to macrophage activation and progression of metabolic dysfunction-associated steatohepatitis (MASH). Experimental data demonstrated that TMEM189 is highly expressed in macrophages, and its inactivation promotes M1 macrophage polarization and increases lipid loading in co-cultured hepatocytes. Loss of *Tmem189* in myeloid cells results in a selective increase in the population and activation of monocyte-derived macrophages (MDMs) in the liver, which exacerbates hepatic steatosis, inflammatory damage, and fibrosis in diet-induced MASH mice. The restoration of Tmem189 expression in hepatic macrophages significantly attenuates MASH-related phenotypes. Mechanistically, TMEM189 interacts with fatty acid synthase (FASN), negatively regulates its expression and pro-inflammatory response in macrophages. Further studies revealed that *TMEM189* deletion enhances FASN-promoted NLRP3 palmitoylation and its activation, leading to increased IL-1β production, which in turn further accelerates MASH progression. Our findings provide important evidence supporting the critical regulatory role of TMEM189-FASN signaling in liver macrophage activation and MASH progression, which may represent a potential therapeutic target for MASH treatment.

## Introduction

Metabolic dysfunction-associated steatotic liver disease affects nearly 25% of adults worldwide, posing a significant public health burden. The disease progresses from metabolic dysfunction-associated steatotic liver (MASL) to its more severe form, metabolic dysfunction-associated steatohepatitis (MASH), a critical transition point that markedly increases the risk of cirrhosis and hepatocellular carcinoma [1, 2]. MASH is characterized by hepatocellular injury, chronic inflammation, and progressive fibrosis, driven by hepatic caloric overload as a primary trigger [3]. This metabolic stress disrupts hepatocyte homeostasis, leading to oxidative damage, inflammatory activation, and microenvironmental remodeling, collectively fueling disease progression [1, 4].

Hepatic macrophages comprise liver-resident Kupffer cells (KCs) and infiltrating monocyte-derived macrophages (MDMs), and they play a pivotal role in propagating inflammation in MASH [5]. The activation of macrophages is triggered by damage-associated molecular patterns (DAMPs) from injured hepatocytes and gut-derived PAMPs (due to increased intestinal permeability) [6]. Activated macrophages secrete pro-inflammatory cytokines (e.g., TNF-α, IL-6, IL-1β) and chemokines (e.g., CCL2). CCL2 recruits MDMs, which further amplify hepatic inflammation and promote insulin resistance, accelerating MASH progression. NLRP3 inflammasome activation is a critical pathway in hepatic macrophages, driving the release of IL-1β and IL-18, which sustain inflammation and hepatocyte injury [7, 8]. The pathogenesis of MASH remains multifactorial and incompletely understood, involving intricate crosstalk between metabolic dysfunction, immune activation, and fibrogenic responses.

Transmembrane protein 189 (TMEM189), also known as plasmanylethanolamine desaturase 1 (PEDS1), is a key enzyme in plasmalogen biosynthesis, catalyzing the formation of the vinyl ether double bond that confers potent antioxidant properties to plasmalogens [9, 10]. Reduced serum plasmalogen levels have been observed in MASH patients, suggesting a possible link between TMEM189 dysregulation and disease progression [11]. In tumor cells, TMEM189 suppresses ferroptosis by destabilizing FAR1, thereby promoting tumor survival and progression [12, 13]. Our previous study revealed that TMEM189 inhibits autophagy by disrupting ULK1 proteostasis, highlighting its role in cellular stress responses [14]. The human TMEM189-UBE2V1 readthrough transcript harbors the most significant SNP associated with COVID-19 severity and is implicated in IL-1 signaling [15]. Bioinformatics analysis (https://www.proteinatlas.org/ENSG00000240849-PEDS1, https://dice-database.org/ genes/TMEM189) indicates that TMEM189 is highly expressed in monocytes/macrophages across multiple tissues (e.g., liver, kidney, stomach, adipose tissues) and blood. Elevated *Tmem189* expression in mouse liver macrophages (https://cells.ucsc.edu/?ds=liver-macrophages+rna-seq&gene=Tmem189&exprGene) and LPS-stimulated mouse bone marrow-derived macrophages (BMDMs) (http://biogps.org/#goto=gene-report&id=407243) was also observed, indicating that TMEM189 may be involved in the regulation of macrophages and inflammatory responses. Data from RNA-seq suggest that *Tmem189* mRNA is increased in hepatic macrophages of MASH mice [16], implicating it in disease-related immune dysfunction. A recent study demonstrates that *Peds1* deficiency resulted in exacerbated inflammation and delayed development in zebrafish larvae [17]. However, the function of TMEM189 in macrophage physiology and MASH pathogenesis remains unknown.

Here, we generated myeloid-specific *Tmem189* knockout (KO) mice and in vitro and in vivo investigated the role of TMEM189-regulated macrophages in inflammatory responses during MASH evolution. Deletion of *Tmem189* expanded and hyper-activated the hepatic macrophage pool, primarily MDMs, thereby sustaining a chronic inflammatory microenvironment in the liver. These primed macrophages released pro-inflammatory factors (e.g., TNF-α, IL-6, IL-1β and CCL2) that directly damaged hepatocytes, accelerated lipid accumulation and amplified hepatic fibrosis. Furthermore, TMEM189 negatively regulates FASN expression and enhances FASN-promoted NLRP3 palmitoylation and its activation. Our studies suggest that targeting TMEM189 may have therapeutic potential for metabolic liver disease including MASH.

## Results

### *Tmem189* KO in macrophages aggravates diet-induced MASH

To investigate the role of TMEM189, we first characterized its expression profile in immune cells from C57BL/6 J wildtype (WT) mice. Quantitative RT-PCR (qRT-PCR) revealed abundant *Tmem189* expression in bone marrow-derived macrophages (BMDMs) and peritoneal macrophages (PMs) (Fig. S1a). We then examined TMEM189 expression in phorbol myristate acetate (PMA)-stimulated human THP-1 macrophages. Compared with unstimulated THP-1 monocytes, *TMEM189* transcription was significantly increased following PMA stimulation (Fig. S1b). We next isolated hepatic macrophages and hepatocytes from WT mouse livers and found that *Tmem189* mRNA was significantly higher in hepatic macrophages than in hepatocytes under normal conditions (Fig. S1c). These findings indicated that TMEM189 is highly expressed in macrophages, which is comparable with the analysis of public databases (https://www.proteinatlas.org/ENSG00000240849-PEDS1, https://dice-database.org/genes/TMEM189, https://cells.ucsc.edu/?ds=liver-macropha-ges+rna-seq&gene=Tmem189&exprGene).

To clarify the functional roles of macrophage-expressed TMEM189 in the inflammatory response, we generated myeloid-specific *Tmem189* conditional knockout (KO) (*Tmem189*^*fl/fl*^*Lyz2*^*Cre*^) mice and their littermates (*Tmem189*^*fl/fl*^) in the C57BL/6 genetic background (Fig. S2a). The *Tmem189* KO was confirmed by performing PCR of mouse tail genomic DNA (Fig. S2b), immunoblotting of BMDMs (Fig. S2d), and quantitative RT-PCR analysis of BMDMs (Fig. S2e) and hepatic macrophages (Fig. S2f). The resulting *Tmem189*^*fl/fl*^*Lyz2*^*Cre*^ mice did not exhibit a spontaneous pathological phenotype compared with age-matched *Tmem189*^*fl/fl*^ mice. Flow cytometry analysis revealed that there was no significant difference in the proportion of macrophages, monocytes, neutrophils, T cells, or B cells **(**Fig. S3) in different tissues and blood between *Tmem189*^*fl/fl*^ and *Tmem189*^*fl/fl*^*Lyz2*^*Cre*^ mice in normal conditions. These results suggest that *Tmem189*-specific depletion in the myeloid cell lineage does not influence the immunocyte development and mouse growth process.

**Fig. 1:**
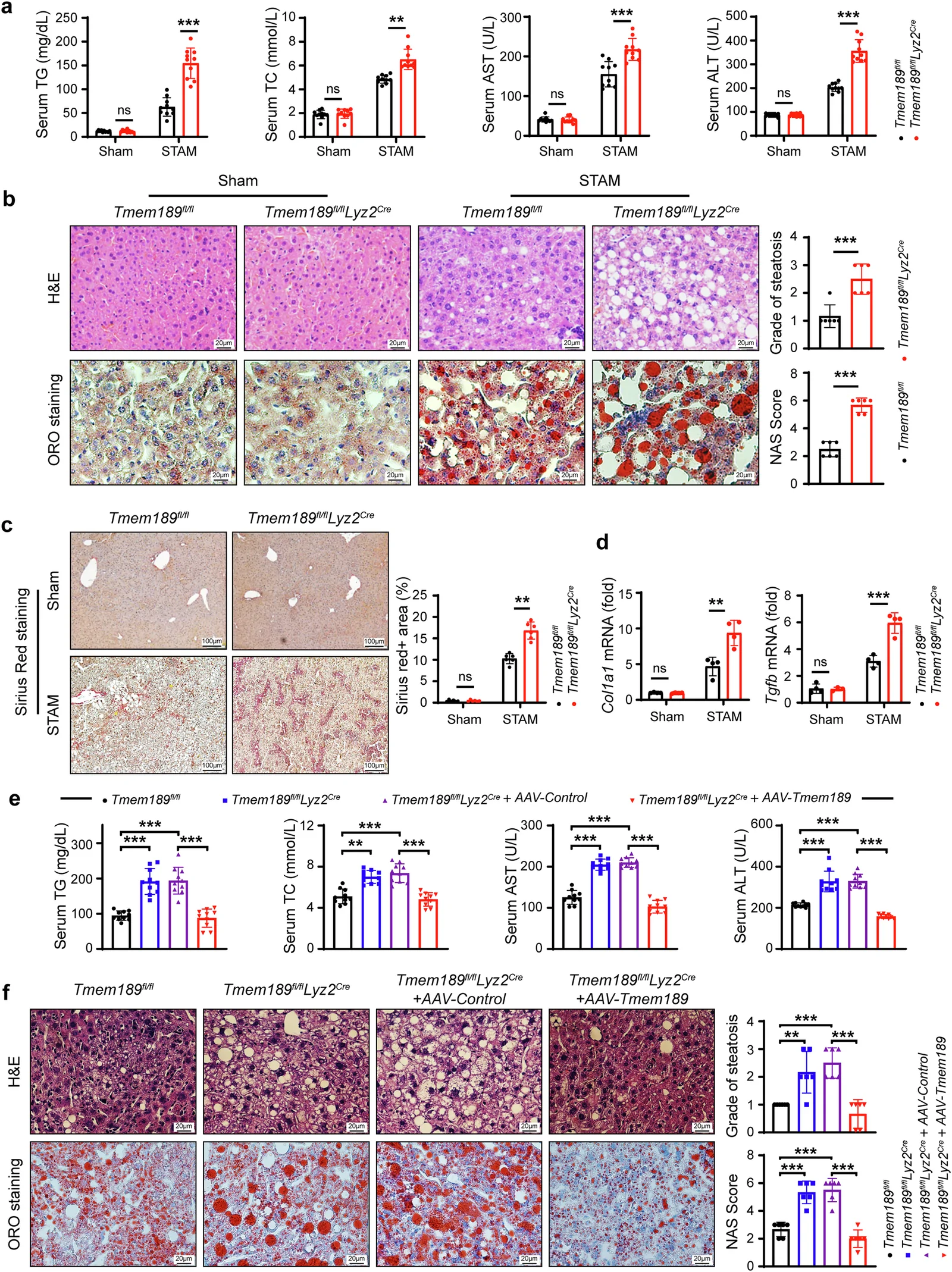
Knockout of *Tmem189* in hepatic macrophages exacerbates diet-induced MASH. **a** Serum levels of TG, TC, AST and ALT were measured in the indicated mice (*n* = 10). **b** Representative images of liver tissues stained with H&E or ORO from different groups. The grade of steatosis and NAS score per field were quantified, respectively. **c** Representative images of liver tissues stained with Sirius Red and the quantification of Sirius Red-positive fields. **d** qRT-PCR analysis of *Col1a1* and *Tgfb* mRNA levels in liver tissues from the indicated mice (*n* = 4). **e** Serum levels of TG, TC, AST, and ALT were measured in STAM mice from different groups (*n* = 10). **f** Representative images of liver tissues stained with H&E or ORO from different groups. The grade of steatosis and NAS score per field were quantified, respectively. Statistical significance was determined using the multiple *t*-test with the two-stage linear step-up procedure of Benjamini, Krieger and Yekutieli (**a**, **c**, **d**), unpaired two-tailed Student’s *t*-test (**b**), and one-way ANOVA followed by Tukey’s honestly significant difference (HSD) post hoc test for multiple comparisons (**e**, **f**). Data are presented as mean ± SD. ns not significant, **p < 0.01, ***p < 0.001.

To investigate the macrophage-specific function of TMEM189 in MASH pathogenesis, we used *Tmem189*^*fl/fl*^ and *Tmem189*^*fl/fl*^ *Lyz2*^*Cre*^ male mice to subject either STAM, HFD or MCD models (Fig. S4a–c). In the STAM model, *Tmem189*^*fl/fl*^*Lyz2*^*Cre*^ mice exhibited significantly exacerbated MASH phenotypes compared to *Tmem189*^*fl/fl*^ controls, as evidenced by elevated serum levels of triglycerides (TG), total cholesterol (TC), ALT, and AST (Fig. 1a). Histological analysis using H&E and Oil Red O (ORO) staining revealed liver tissue disorganization, extensive hepatocyte ballooning, and marked lipid droplet accumulation in *Tmem189*^*fl/fl*^*Lyz2*^*Cre*^ mice (Fig. 1b). Similar phenotypic alterations were observed in the HFD- or MCD- fed cohort, in which *Tmem189*^*fl/fl*^*Lyz2*^*Cre*^ mice demonstrated comparable pathological changes to those observed in the STAM model (Figs. S5, 6). To assess the contribution of TMEM189 to the development of hepatic fibrosis, we performed quantitative analysis of fibrotic markers. *Tmem189*^*fl/fl*^*Lyz2*^*Cre*^ STAM mice displayed a significant increase in Sirius Red staining area compared to control littermates (Fig. 1c). Furthermore, upregulation of profibrotic marker genes *Col1a1* and *Tgfb1* was also observed in *Tmem189*^*fl/fl*^*Lyz2*^*Cre*^ STAM mice (Fig. 1d), indicating worsening of the MASH phenotype. These findings demonstrated that myeloid-specific *Tmem189* KO accelerates both hepatic steatosis and fibrosis progression in diet-induced MASH models.

We also generated Kupffer cell-specific *Tmem189* KO (*Tmem189*^*fl/fl*^*Clec4f*^*Cre*^) mice (Fig. S2a, c, g) to investigate the role of TMEM189 in KCs. *Tmem189*^*fl/fl*^*Clec4f*^*Cre*^ mice and *Tmem189*^*fl/fl*^ control littermates were subjected to the STAM model. As shown in Fig. S7, there was no significant difference in pathological phenotypes between *Tmem189*^*fl/fl*^ and *Tmem189*^*fl/fl*^*Clec4f*^*Cre*^ mice, suggesting that KCs may not be involved in the MASH pathogenesis promoted by *Tmem189* knockout.

To determine whether TMEM189 restoration in hepatic macrophages could suppress the pathological phenotypes resulting from its deletion, we performed gain-of-function experiments. *Tmem189*^*fl/fl*^*Lyz2*^*Cre*^ male mice received *AAV8*-mediated *Tmem189* delivery under the macrophage-specific F4/80 promoter and were subjected to the STAM model (Fig. S4d). Immunofluorescence assays confirmed the overexpression of TMEM189 in hepatic F4/80^+^ macrophages (Fig. S8). Data from experiments proved that TMEM189 restoration in *Tmem189*^*fl/fl*^*Lyz2*^*Cre*^ STAM mice significantly ameliorate the liver damage and metabolic dysfunction, as evidenced by reduced serum TG, TC, AST, and ALT levels (Fig. 1e). Furthermore, hepatic steatosis was substantially attenuated following TMEM189 reconstitution (Fig. 1f). Collectively, these findings demonstrate that elevated TMEM189 expression in hepatic macrophages exerts a protective function against MASH progression.

### *Tmem189* KO increases the population of monocyte-derived macrophages (MDMs) in diet-induced MASH livers

**Fig. 2:**
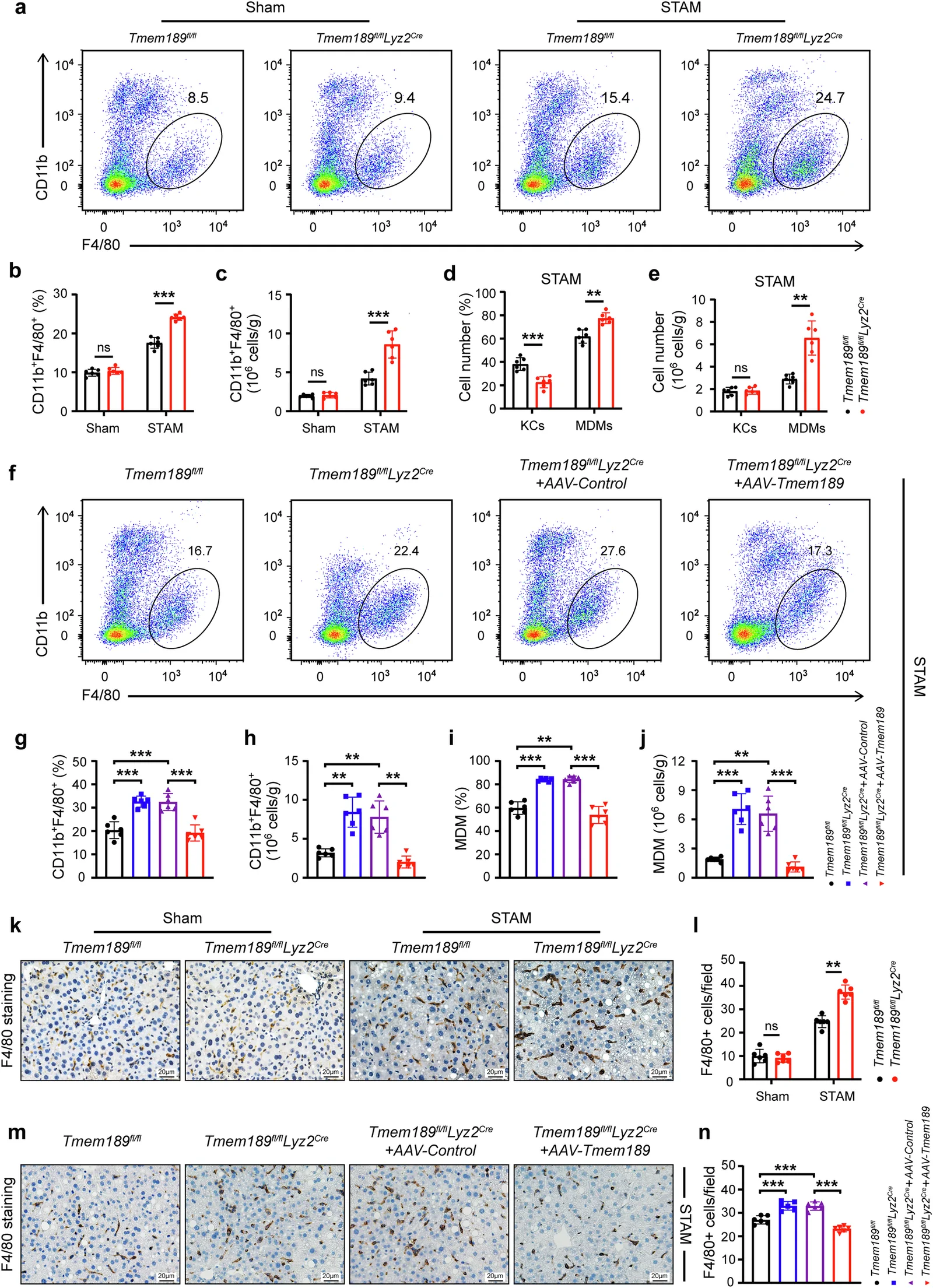
*Tmem189* KO increases the abundance of MDMs rather than KCs in diet-induced MASH mice. **a** Representative images of flow cytometry analysis to hepatic macrophages (CD45^+^Ly6G^-^CD11b^+^F4/80^+^) in indicated mice. **b**, **c** The percentage and total number of hepatic macrophages were quantified by flow cytometry analysis in indicated mice (*n* = 6). **d**, **e** The percentage and total number of MDMs (CD45^+^Ly6G^-^CD11b^+^F4/80^+^Tim4^-^) or KCs (CD45^+^Ly6G-CD11b^+^F4/80^+^Tim4^+^) in the indicated mice were detected by flow cytometry (*n* = 6). **f** Representative images of flow cytometry analysis to hepatic macrophages (CD45^+^Ly6G^-^CD11b^+^F4/80^+^) in indicated STAM mice. **g**, **h** The percentage and total number of hepatic macrophages were quantified by flow cytometry analysis (*n* = 6). **i**, **j** The percentage and total number of MDMs were quantified by flow cytometry (*n* = 6). **k**–**n** Representative images of F4/80 immunohistochemistry staining in different mouse livers (**k**, **m**). The number of F4/80^+^ cells per field was quantified (**l** and **n**) by ImageJ. Statistical significance was determined using the multiple *t*-test with the two-stage linear step-up procedure of Benjamini, Krieger and Yekutieli (**b**–**e**, **l**) and one-way ANOVA followed by Tukey’s honestly significant difference (HSD) post hoc test for multiple comparisons (**g**–**j**, **n**). Data are presented as mean ± SD. ns not significant, **p < 0.01, ***p < 0.001.

It is widely recognized that the activation of macrophages is crucial for initiating and exacerbating hepatic inflammation, which contributes to the progression from simple steatosis to MASH [18]. Consequently, we sought to further define the role of Tmem189 in hepatic macrophages within the context of MASH. Through flow cytometry analysis, we analyzed total hepatic macrophages (CD45^+^Ly6G^-^CD11b^+^F4/80^+^), KCs (CD45^+^Ly6G^-^CD11b^+^F4/80^+^Tim4^+^) and MDMs (CD45^+^Ly6G^-^CD11b^+^F4/80^+^Tim4^-^) from various mouse livers based on the gating strategy (Fig. S9a). In the STAM mouse model, the deletion of *Tmem189* significantly increased the percentage and absolute numbers of hepatic macrophages per gram, compared to control mice (Fig. 2a–c). Similar results were obtained from HFD mouse model (Fig. S9b, c). Further analysis revealed that the increased hepatic macrophages in *Tmem189*^*fl/fl*^*Lyz2*^*Cre*^ STAM or HFD mice were primarily MDMs, as evidenced by the elevated percentage and absolute numbers of MDMs per gram (Fig. 2d, e, Fig. S9d). However, the percentage of KCs was decreased, while the total number of KCs remained unchanged (Fig. 2d, e, Fig. S9d). Comparable results were observed in MCD mice (Fig. S9e–g). Upon restoration of Tmem189 in hepatic macrophages of *Tmem189*^*fl/fl*^*Lyz2*^*Cre*^ STAM mice, the percentage and absolute numbers of hepatic macrophages per gram were decreased (Fig. 2f–h**)**. Accordingly, the percentage and absolute numbers of MDMs in Tmem189-restored *Tmem189*^*fl/fl*^*Lyz2*^*Cre*^ STAM mice were also reduced (Fig. 2i,j)*.* In line with the flow cytometry results, immunohistochemistry assays confirmed that the accumulation of F4/80^+^ macrophages was also elevated in *Tmem189* KO mice (Fig. 2k, l, Fig. S10a, b). The reintroduction of *Tmem189* led to a reduced number of F4/80^+^ macrophages in *Tmem189*^*fl/fl*^*Lyz2*^*Cre*^ STAM mice (Fig. 2m, n). Additionally, *Tmem189* deletion did not affect the percentage and absolute numbers per gram of monocytes or neutrophils within the liver tissues in diet-induced MASH mice with different genetic backgrounds (data not shown). These findings above suggest that *Tmem189*-depleted hepatic MDMs are the key contributors to the advancement of MASH.

### *Tmem189* deletion promotes the activation of macrophages in diet-induced MASH livers

**Fig. 3:**
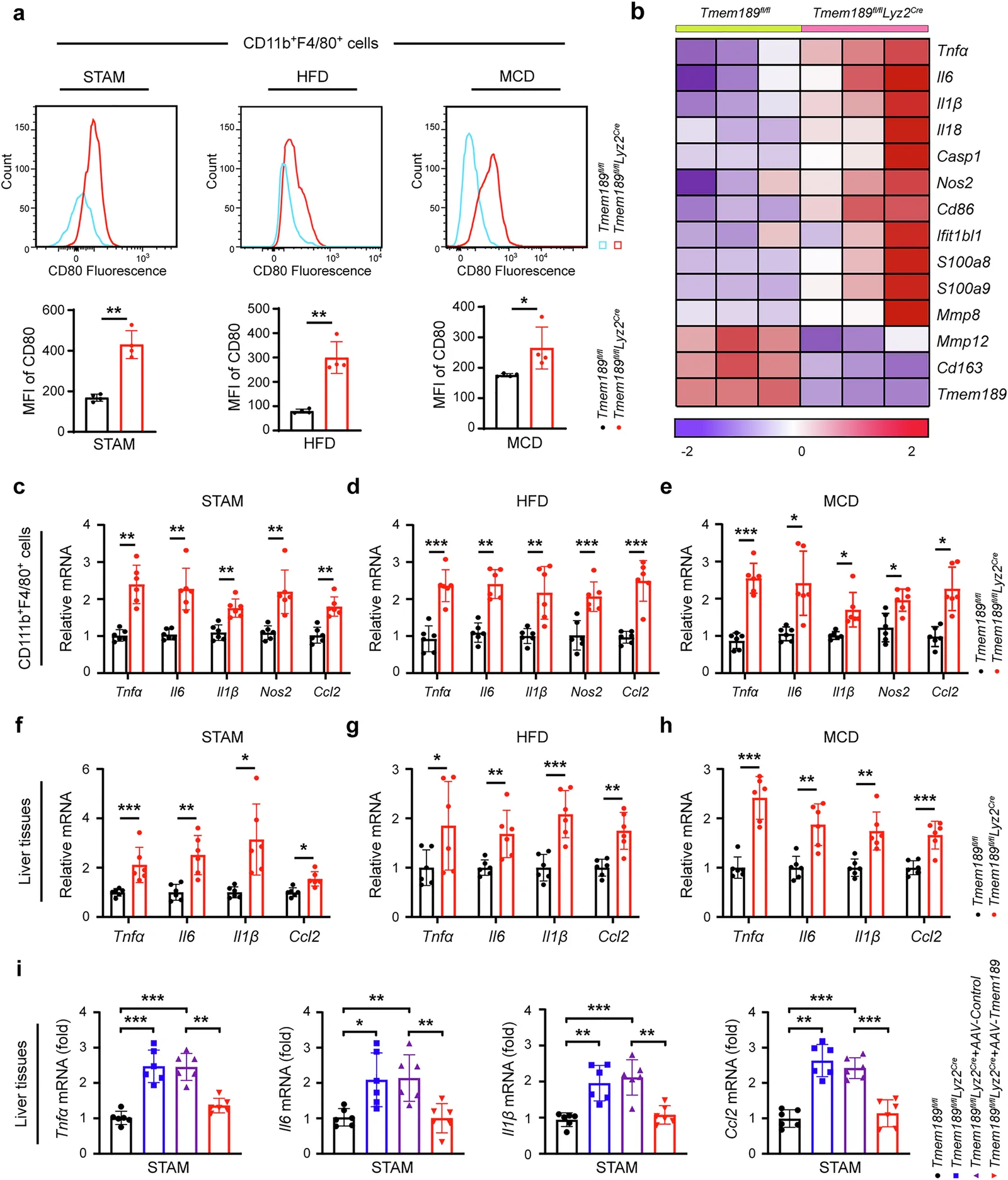
*TMEM189* deficiency aggravates hepatic macrophage activation and pro-inflammatory liver microenvironment. **a** The mean fluorescence intensity (MFI) of CD80 expression in hepatic macrophages from different model groups was assessed by flow cytometry and quantified (*n* = 4). **b** Hepatic macrophages (CD45^+^Ly6G^-^CD11b^+^F4/80^+^) were isolated and purified from male *Tmem189*^*fl/fl*^ or *Tmem189*^*fl/fl*^*Lyz2*^*Cre*^ STAM mice. A hierarchical clustering map of total mRNA was shown (*n* = 3). **c**–**e** The mRNA levels of *Tnfα*, *Il6*, *Il1β*, *Nos2* and *Ccl2* in hepatic macrophages from the indicated mice were detected by qRT-PCR (*n* = 6). **f**–**h** The mRNA levels of *Tnfα*, *Il6*, *Il-1β* and *Ccl2* in liver tissues from different models were measured by qRT-PCR (*n* = 6). **i** The mRNA levels of *Tnfα*, *Il6*, *Il-1β* and *Ccl2* in liver tissues from different groups were measured by qRT-PCR (*n* = 6). Statistical significance was determined using the unpaired two-tailed Student’s *t*-test (**a**), multiple *t*-test with the two-s*t*age linear step-up procedure of Benjamini, Krieger and Yekutieli (**c**–**h**), and one-way ANOVA followed by Tukey’s honestly significant difference (HSD) post hoc test for multiple comparisons (**i**). Data are presented as mean ± SD. *p < 0.05, **p < 0.01, ***p < 0.001.

We further investigated the activation of isolated hepatic macrophages from various mouse livers. As depicted in Fig. 3a, the expression of CD80 fluorescence signals in CD11b^+^F4/80^+^ cells was increased in *Tmem189* KO macrophages among the livers of STAM, HFD, or MCD mice. Transcriptomics results indicated that in isolated hepatic macrophages from *Tmem189* KO STAM mice, the mRNA levels of pro-inflammatory cytokines were obviously elevated (Fig. 3b). Data from qRT-PCR confirmed that in isolated *Tmem189* KO hepatic macrophages, the mRNA levels of *Tnfα*, *Il6*, and *Il-1β*, chemokine *Ccl2*, as well as *Nos2* mRNA, were significantly upregulated in MASH model mice (Fig. 3c–e). These findings suggest that the deletion of *Tmem189* enhances the activation of hepatic macrophages in diet-induced MASH.

Additionally, the levels of pro-inflammatory factors in the liver microenvironment were also measured in different mice. As illustrated in Fig. 3f–h, the mRNA levels of pro-inflammatory cytokines such as *Tnfα, Il6*, and *Il1β*, as well as the chemokine *Ccl2* in liver tissue lysates, were increased in *Tmem189* KO STAM, HFD, or MCD mice compared with control mice. However, restored TMEM189 expression in hepatic macrophages could decrease the transcript of these inflammatory mediators in *Tmem189*^*fl/fl*^*Lyz2*^*Cre*^ STAM livers (Fig. 3i). Collectively, these results demonstrated that increased expression of TMEM189 in hepatic macrophages could alleviate liver inflammatory damage and diet-induced MASH progression.

### *Tmem189* loss promotes macrophage polarization towards M1 phenotype and enhances lipid accumulation in hepatocytes

**Fig. 4:**
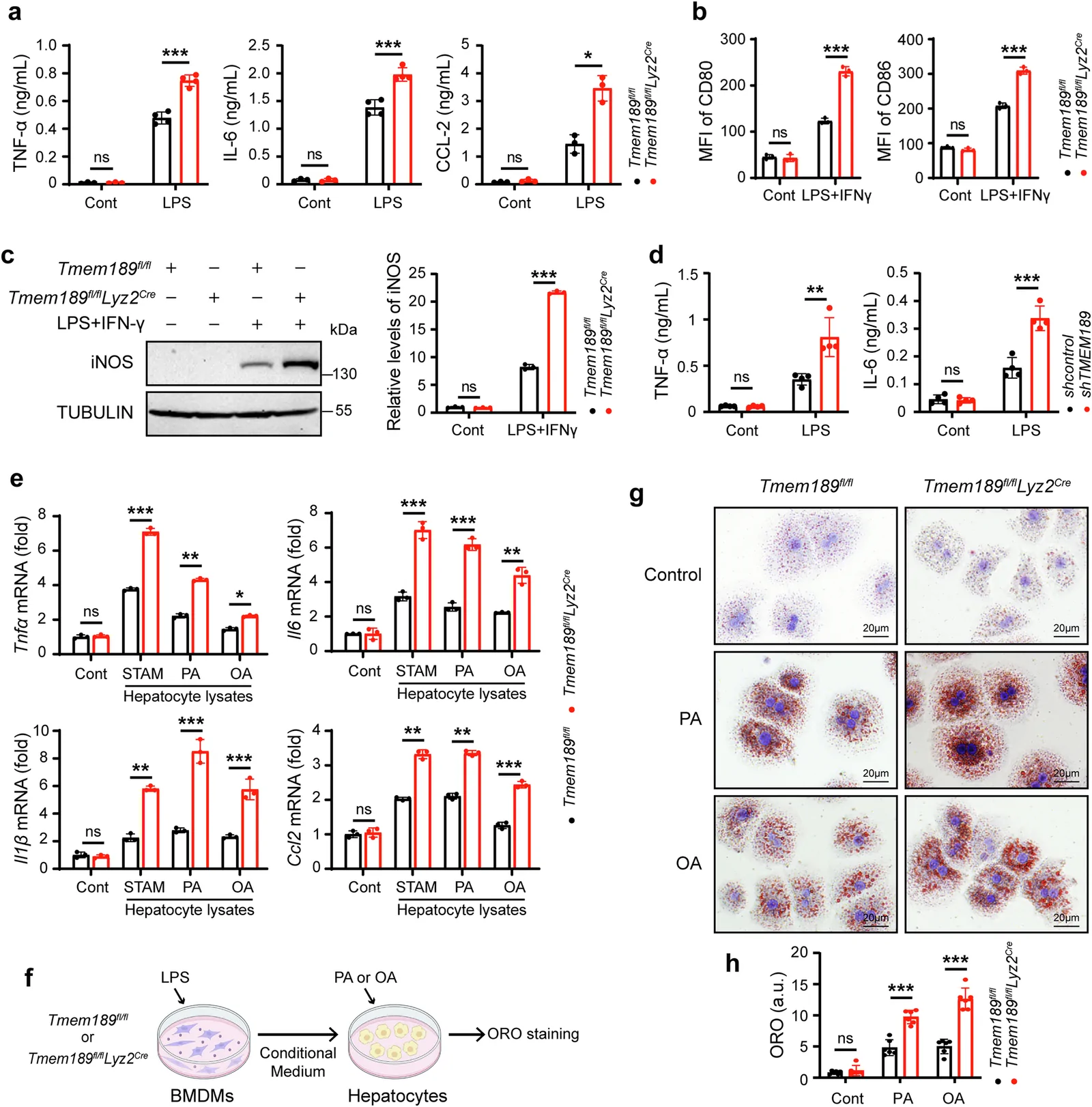
Loss of *TMEM189* drives macrophages M1 polarization and exacerbates hepatocytes steatosis. **a** BMDMs from *Tmem189*^*fl/fl*^ or *Tmem189*^*fl/fl*^*Lyz2*^*Cre*^ mice were stimulated with LPS (1 μg/mL) for 24 hours. The secretion of TNF-α, IL-6, and CCL-2 in culture supernatant was detected by ELISA assay. **b** BMDMs from *Tmem189*^*fl/fl*^ or *Tmem189*^*fl/fl*^*Lyz2*^*Cre*^ mice were stimulated with LPS (1 μg/mL) plus IFN-γ (20 ng/mL) for 24 hours. The MFI of CD80 or CD86 expression was quantified by flow cytometry and quantified. **c** BMDMs were treated as in (**b**), the levels of iNOS were determined by western blotting. The amounts of iNOS relative to TUBULIN were quantified, with the average value in *Tmem189*^*fl/fl*^ BMDMs without LPS/IFN-γ treatment normalized to 1. **d** PMA-differentiated *shcontrol* or s*hTMEM189* THP-1 cells were stimulated with LPS (1 μg/mL) for 24 hours. The secretion of TNF-α and IL-6 in culture supernatant was detected by ELISA assay. **e** Primary hepatocytes isolated from STAM mice or from wildtype mice (the latter treated with 0.4 mM PA, or 0.2 mM OA for 8 hours) were lysed via freeze-thaw cycles. These cell lysates and BSA (control) were subsequently co-cultured with BMDMs obtained from *Tmem189*^*fl/fl*^ or *Tmem189*^*fl/fl*^*Lyz2*^*Cre*^ mice. The transcripts of *Tnfα, Il6, Il1β*, and *Ccl2* were analyzed using qRT-PCR. **f** Schematic illustration of the experimental procedure. BMDMs from *Tmem189*^*fl/fl*^ or *Tmem189*^*fl/fl*^*Lyz2*^*Cre*^ mice were stimulated with LPS (1 μg/mL) for 24 hours. The conditioned medium was then collected and co-cultured with primary hepatocytes isolated from normal wildtype mice for 12 hours. These hepatocytes were subsequently treated with PA (0.4 mM) or OA (0.2 mM) for 8 hours and subjected to Oil Red O (ORO) staining. **g** Representative images of ORO staining in primary hepatocytes. **h** Quantitative analysis of ORO staining by ImageJ software. Statistical significance was determined using the multiple *t*-test with the two-stage linear step-up procedure of Benjamini, Krieger and Yekutieli (**a**–**e**, **g**). All plots represent the mean ± SD. ns, not significant, *p < 0.05, **p < 0.01, ***p < 0.001. Experiments were repeated at least three times with similar results.

During the progression of MASH, increased intestinal permeability leads to greater exposure of hepatic macrophages to gut-derived bacterial products. Among these, lipopolysaccharide (LPS), a major bacterial endotoxin, is known to activate hepatic macrophages in vivo [19]. Therefore, we examined the response of *Tmem189* KO macrophages to LPS stimulation in vitro. Consistent with the in vivo findings, LPS-treated *Tmem189-*null BMDMs exhibited markedly elevated mRNA levels of pro-inflammatory cytokines (*Tnfα, Il6*, and *Il1β*) and the chemokine *Ccl2* at various time points (Fig. S11a). ELISA further confirmed the increased secretion of TNFα, IL-6, and CCL2 proteins into the culture medium following LPS challenge (Fig. 4a). Flow cytometry analysis revealed the enhanced surface expression of CD80 and CD86 in *Tmem189*-deleted BMDMs stimulated with LPS/IFN-γ (Fig. 4b), while western blotting showed elevated intracellular iNOS levels (Fig. 4c). Collectively, these results demonstrate that *Tmem189* KO sensitizes macrophages to LPS and polarizes them toward an M1-like phenotype.

We subsequently expanded our research to a human macrophage model by creating stable *TMEM189*-knockdown THP-1 cells using lentiviral delivery of *pLVX-shTMEM189* (*shTMEM189*). The successful knockdown of *TMEM189* in THP-1 cells was confirmed by western blotting and qRT-PCR assays (Fig. S11b, c). Upon exposure to LPS, the *TMEM189*-silenced cells exhibited heightened pro-inflammatory responses, showing significantly elevated transcript levels of *TNF-α, IL-6, IL-11β, and CCL-2* (Fig. S11d) and secreting increased amounts of TNF-α and IL-6 proteins (Fig. 4d). This alignment with data from mouse *Tmem189*-null BMDMs strengthens the conclusion that the loss of TMEM189 function predisposes macrophages to over-activation, indicating a novel anti-inflammatory role for macrophage TMEM189 in innate immunity.

During MASH progression, injured hepatocytes release damage-associated molecular patterns (DAMPs), which contribute to disease exacerbation by activating macrophages. To model this process in vitro (Fig. S12), we collected primary hepatocytes from various treatments and subjected them to freeze-thaw cycles to prepare cell lysates, which were then incubated with mouse BMDMs. qRT-PCR analysis revealed that, compared to *Tmem189*^*fl/fl*^ BMDMs, *Tmem189* KO BMDMs exhibited significantly higher mRNA levels of the pro-inflammatory mediators (*Tnfα, Il6, Il1β* and *Ccl2*) (Fig. 4e). This demonstrates that *Tmem189* deletion potentiates macrophage activation in response to signals from injured hepatocytes, both in vitro and in vivo.

We further investigated whether activated macrophages affect hepatocyte lipid accumulation in vitro. Conditioned media were collected from LPS-stimulated BMDMs from different genotypes and utilized to treat primary mouse hepatocytes (Fig. 4f). Following the induction of steatosis with palmitic acid (PA) or oleic acid (OA), Oil Red O staining showed that the *Tmem189* KO group had more abundant lipid droplet accumulation in primary hepatocytes compared to the *Tmem189*^*fl/fl*^ group (Fig. 4g). This effect was replicated in human cells: PLC/PRF/5 hepatoma cells exposed to conditioned media from *TMEM189*-knockdown THP-1 macrophages also developed more lipid droplets than those in the *shcontrol* group (Fig. S11e–g). These data suggest that the activation of *TMEM189*-deleted macrophages enhance their ability to promote lipid accumulation in hepatocytes.

**Fig. 5:**
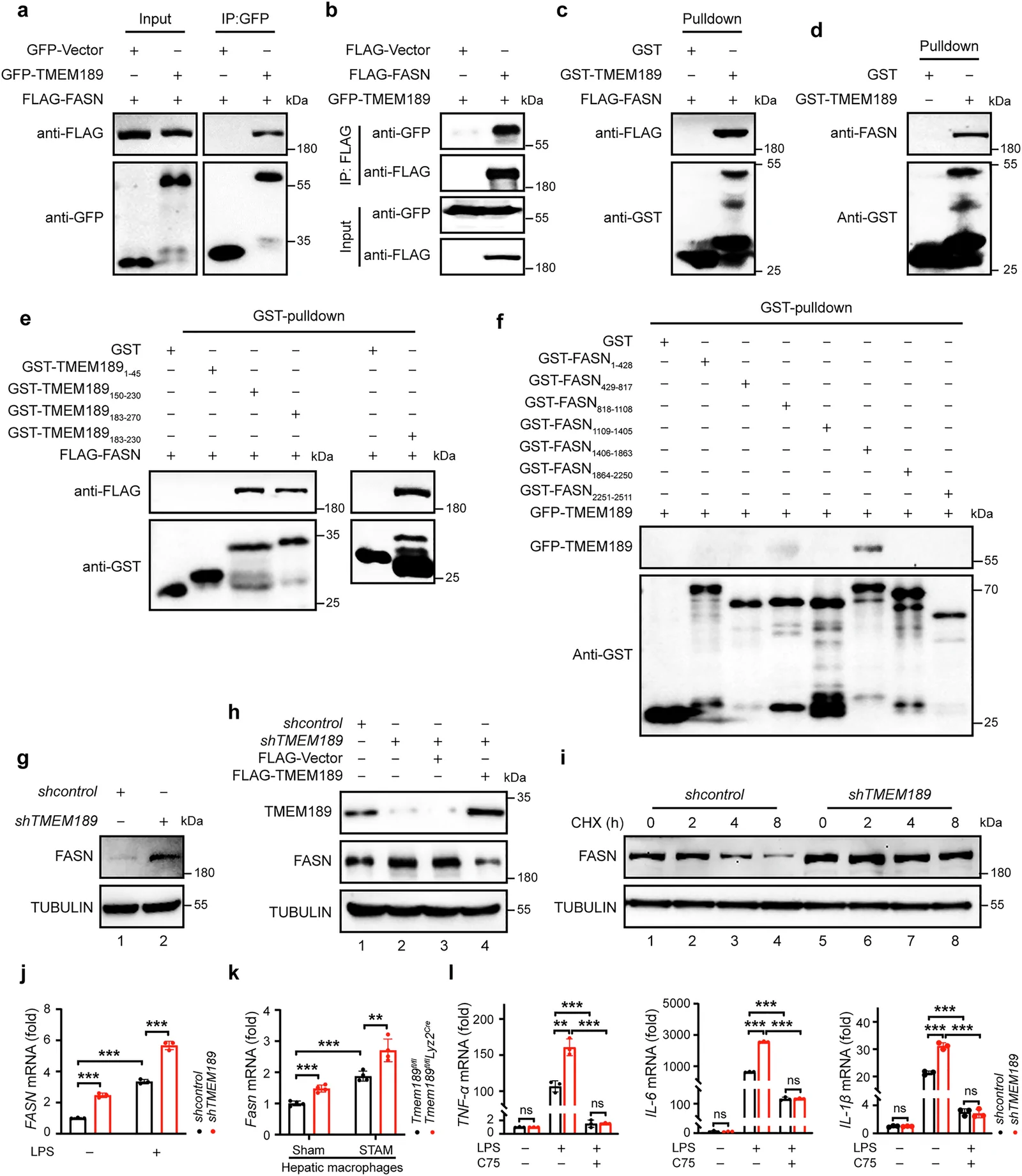
TMEM189 interacts with FASN and negatively regulates its expression and pro-inflammatory activities. **a**, **b** HEK293T cells were co-tr**a**nsfected with the indicated plasmids for 24 hours. Cell lysates were subjected to IP using anti-GFP or anti-FLAG antibodies, and the indicated proteins were detected in the immunoprecipitates by western blotting. Simultaneously, 10% of the cell lysates were used for immunoblotting. Recombinant GST-TMEM189 and GST proteins were purified and immobilized on Glutathione-Sepharose beads, then incubated with HEK293T cell lysates containing FLAG-FASN (**c**) or THP-1 cell lysates (**d**). Proteins retained on the washed beads were then immunoblotted using the indicated antibodies. Recombinant GST and the indicated GST-fusion proteins were incubated with HEK293T cell lysates containing FLAG-FASN (**e**) or GFP-TMEM189 (**f**), and proteins retained on the washed beads were immunoblotted using the indicated antibodies. **g** The levels of FASN protein in PMA-differentiated *shcontrol* or *shTMEM189* THP-1 cells were measured by western blotting. **h** Stable *shTMEM189*-expressing THP-1 cells were transfected with the indicated plasmids for 24 hours. The levels of FASN protein were analyzed by western blotting. **i** PMA-differentiated *shcontrol* or *shTMEM189* THP-1 cells were treated with CHX for 0, 2, 4, or 8 hours. The levels of FASN protein were detected by western blotting. (**j**) qRT-PCR analysis of the *FASN* transcript in *shcontrol* or *shTMEM189* THP-1 cells stimulated with or without LPS for 12 hours. **k** Hepatic macrophages were isolated from *Tmem189*^*fl/fl*^ or *Tmem189*^*fl/fl*^*Lyz2*^*Cre*^ Sham or STAM mice; the levels of *Fasn* mRNA were analyzed by qRT-PCR. (**l**) *shcontrol* or *shTMEM189* THP-1 cells were pretreated with or without the FASN inhibitor C75 for 6 hours, then stimulated with LPS for 12 hours. The mRNA levels of *TNF-α*, *IL-6*, and *IL-1β* were quantified by qRT-PCR. Statistical significance was determined using the multiple *t*-test with the two-stage linear step-up procedure of Benjamini, Krieger and Yekutieli and one-way ANOVA followed by Tukey’s honestly significant difference (HSD) post hoc test for multiple comparisons (**j**–**l**). All plots represent the mean ± SD. ns not significant, **p < 0.01, ***p < 0.001. Experiments were repeated at least three times with similar results.

### TMEM189 interacts with fatty acid synthase (FASN), negatively regulates its expression and pro-inflammatory activities

We subsequently conducted a series of experiments to clarify the molecular mechanism underlying TMEM189’s role in macrophage activation. Data from Immunoprecipitation-Mass Spectrometry (IP-MS) revealed that TMEM189 interacts with fatty acid synthase (FASN). Further co-immunoprecipitation (Co-IP) assays confirmed the presence of the FLAG-FASN protein within GFP-TMEM189 immunoprecipitates (Fig. 5a). Conversely, GFP-TMEM189 protein was also detected in FLAG-FASN immunoprecipitates (Fig. 5b). GST-pulldown experiments further demonstrated that GST-TMEM189 could bind both overexpressed FLAG-FASN and endogenous FASN in THP-1 cells (Fig. 5c, d). These results substantiate the interaction between TMEM189 and FASN within cells.

Furthermore, we created several truncations of GST-TMEM189 based on its functional domains (Fig. S13a) and investigated which TMEM189 domain was responsible for the FASN interaction. Data from GST-pulldown experiments indicated that the amino acid region 183-230 of TMEM189 is sufficient to interact with FASN (Fig. 5e). Subsequently, we constructed seven truncates of GST-FASN (Fig. S13b), and discovered that the amino acid domain 1406-1863 of FASN was responsible for the interaction with GFP-TMEM189 (Fig. 5f). Collectively, these data suggest that FASN is a physiological and direct binding partner of TMEM189.

We further investigated the biological significance of the TMEM189-FASN interaction. In PMA-differentiated THP-1 cells, knockdown of *TMEM189* resulted in the up-regulation of endogenous FASN protein compared to control cells (Fig. 5g). Conversely, overexpression of TMEM189 reduced the levels of FASN protein in PMA-differentiated *shTMEM189* THP-1 cells (Fig. 5h, **lane 4 vs. lane 2**). We further analyzed the half-life of the FASN protein using the protein translation inhibitor cycloheximide (CHX). As shown in Fig. 5i, in the absence of TMEM189, the degradation of endogenous FASN protein in THP-1 cells was delayed during CHX treatment, indicating that TMEM189 might negatively regulate the stability of the FASN protein. Simultaneously, in PMA-differentiated THP-1 cells, TMEM189 knockdown also increased the levels of *FASN* mRNA with or without LPS treatment (Fig. 5j). Comparable elevation in *Fasn* transcription was also observed in *Tmem189*-dificient hepatic macrophages (Fig. 5k), with the underlying mechanism requiring further investigation.

Some documents report that FASN facilitates TLR4 signal transduction and proinflammatory macrophage activation [20, 21]. We therefore asked whether TMEM189 regulates macrophage activation via FASN signaling. Data from experiments indicated that pharmacologic blockade of FASN with C75 (a FASN inhibitor) reduced the transcription of pro-inflammatory cytokines (*TNF-α, IL-6*, and *IL-1β*) in LPS-primed macrophages (Fig. 5l), confirming the pro-inflammatory role of FASN. Importantly, we found that treatment with the C75 abrogated the difference in cytokine production between *shcontrol* or *shTMEM189* THP-1 cells under the same conditions (Fig. 5l), indicating that the hyper-inflammatory phenotype of *TMEM189*-deficient macrophages depends on FASN activity. Thus, TMEM189 helps keep macrophage activation in check, at least in part by downregulating FASN signaling.

### *TMEM189* depletion promotes FASN-regulated NLRP3 palmitoylation and activation

FASN catalyzes the de novo synthesis of endogenous fatty acids, including palmitate, which serves as the essential substrate for protein palmitoylation. This post-translational modification, particularly of NLRP3, is critical for the activation of the NLRP3 inflammasome [22, 23]. We next analyzed the status of inflammasomes in *TMEM189*-deficient macrophages. As shown in Fig. S14a, in *Tmem189*-null BMDMs incubated with either LPS/nigericin or LPS/ATP, the cleavage of caspase-1 and GSDMD N-terminal (GSDMD N) was significantly increased compared with control cells. Similar results were shown in *TMEM189* knockdown PMA-THP-1 cells (Fig. S14b). Consequently, the results of ELISA demonstrated that during the same treatment, mature IL-1β secretion was upregulated in *TMEM189*-ableted macrophages (Fig. S14c, d). Thus, loss of *TMEM189* sensitizes macrophages to NLRP3-dependent inflammasome activation.

**Fig. 6:**
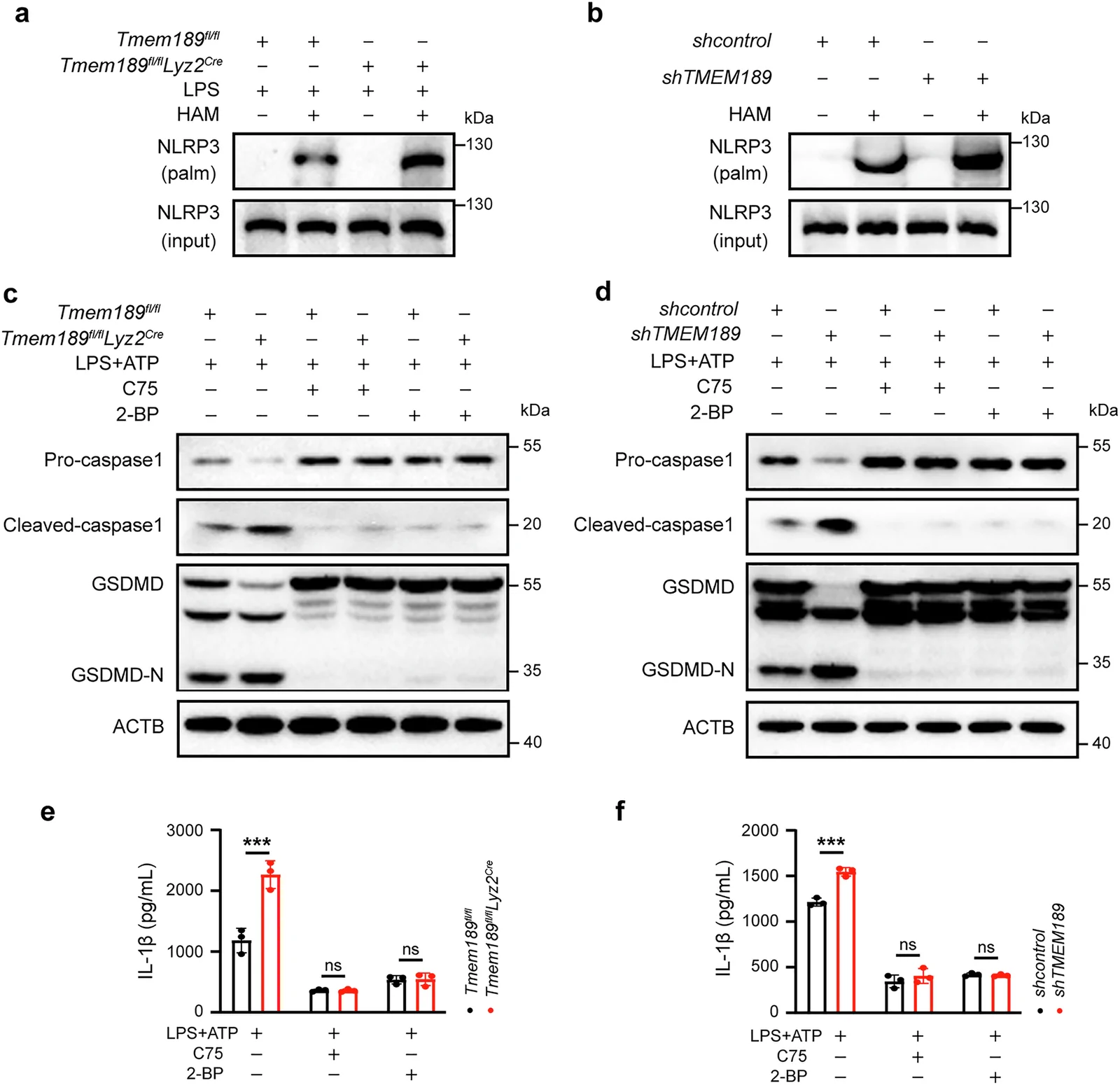
Deletion of *TMEM189* enhances NLRP3 inflammasome activation by increasing FASN-regulated palmitoylation. **a** BMDMs from *Tmem189*^*fl/fl*^ or *Tmem189*^*fl/fl*^*Lyz2*^*Cre*^ mice were primed with LPS (1 μg/mL) for 3 hours, and the protein samples were divided into HAM (+) and HAM (-) samples. The levels of palmitoylated NLRP3 and total NLRP3 (input control) were analyzed by ABE assay and immunoblot analysis. **b** PMA-differentiated *shcontrol* or *shTMEM189* THP-1 cells were lysed and then subjected to ABE assay to evaluate NLRP3 palmitoylation. **c** BMDMs were primed with LPS (1 μg/mL) for 3 hours, stimulated with FASN inhibitor C75 (25 μmol/L) or 2-BP (50 μmol/L) for 2 hours, and finally exposed to ATP (3 mmol/L) for 60 min. Cell lysates were immunoblotted with the indicated antibodies. **d** PMA-differentiated *shcontrol* or *shTMEM189* THP-1 were treated as (**c**), and cell lysates were immunoblotted with the indicated antibodies. The production of IL-1β in the culture supernatants was measured by ELISA. Statistical significance was determined using the multiple *t*-test with the two-stage linear step-up procedure of Benjamini, Krieger and Yekutieli (**e**, **f**). All plots represent the mean ± SD. ns not significant, ***p < 0.001. Experiments were repeated at least three times with similar results.

We subsequently investigated whether *TMEM189* deletion regulates NLRP3 activation by enhancing its palmitoylation, and performed an acyl-biotin exchange (ABE) assay. As shown in Fig. 6a, the presence of a signal upon hydroxylamine (HAM) treatment indicated that endogenous NLRP3 in BMDMs undergoes palmitoylation via a thioester linkage. *Tmem189* KO significantly increased the palmitoylation of NLRP3 protein. Similar results were observed in *TMEM189*-silenced PMA-THP-1 cells (Fig. 6b).

To determine if the FASN pathway regulates NLRP3 palmitoylation in *TMEM189*-deleted macrophages, we inhibited FASN activity by exposing cells to C75. As expected, pretreatment with C75 reduced caspase-1 activation and GSDMD N fragment levels compared to control cells in response to LPS/ATP stimulation (Fig. 6c). At the same time, pretreatment with 2-bromopalmitate (2-BP, a competitive analog of palmitate) also resulted in a similar decrease in caspase-1 cleavage and GSDMD N expression in *Tmem189* KO BMDMs (Fig. 6c). Similar findings were observed in *TMEM189* knockdown PMA-THP-1 cells under the same conditions (Fig. 6d). Accordingly, secreted IL-1β levels decreased upon exposure to C75 or 2-BP in *TMEM189*-deleted macrophages (Fig. 6e, f). Collectively, these results suggest that *TMEM189* deletion-induced activation of the NLRP3 inflammasome may be attributed to FASN-enhanced NLRP3 palmitoylation, thereby highlighting the importance of TMEM189-FASN signaling in modulating macrophage-mediated inflammatory responses.

## Discussion

In the present study, we identified a previously unrecognized anti-inflammatory effects in which macrophage-expressed TMEM189/PEDS1 restrains diet-induced MASH. The macrophage-specific deletion of *Tmem189* expanded and hyperactivated the hepatic MDMs pool, enhancing the expression of TNF-α, IL-6, CCL2, and iNOS, thereby amplifying liver injury in diet-induced MASH mice. Mechanistically, TMEM189 physically engages with FASN; this interaction destabilizes FASN and attenuates downstream inflammatory signaling. Additionally, the protective effect of TMEM189 may contribute to the inactivation of the NLRP3 inflammasome by reducing FASN-enhanced palmitoylation in macrophages, thereby limiting IL-1β release and inflammatory damage. Collectively, our data for the first establish TMEM189 as a critical negative regulator of macrophage activation and chronic hepatic inflammation in the context of MASH.

In the setting of MASH, macrophages can exhibit remarkable plasticity in responding to the signals within the liver tissues and develop into multifaceted phenotypes [24–26]. Liver macrophages consist of two distinct cell populations: self-renewing Kupffer cells (KCs), which originate from yolk sac-derived erythromyeloid progenitors [27, 28], and infiltrating monocyte-derived macrophages (MDMs), which originate from hematopoietic stem cells in the bone marrow [29]. KCs play a major role in maintaining hepatic microenvironmental homeostasis [30, 31]. In the early stages of MASH, they mount certain immune responses to hepatocyte stress and exert immunomodulatory functions. During disease progression, MDMs become the primary drivers of inflammation. They secrete large quantities of inflammatory cytokines such as TNF-α, IL-1β, and IL-6, which exacerbate liver inflammation and hepatocellular injury [32–34]. Yet, the details of liver macrophage regulation and their plasticity in MASH pathogenesis remain to be further defined. Here, our findings demonstrate that *Tmem189* deficiency increases the population of hepatic macrophages and promotes their activation and polarization towards the M1 phenotype, rendering these cells more sensitive to both LPS and signals from damaged hepatocytes. Specifically, *Tmem189*-null MDMs, rather than KCs, exacerbate MASH progression, suggesting that the loss of *TMEM189* in hepatic MDMs drives diet-induced MASH advancement. In vitro, *Tmem189*-null macrophages secrete inflammatory factors that directly promote hepatocyte steatosis in co-culture, establishing an extra-paracrine loop that links macrophage activation to lipid accumulation, thereby providing further evidence of the correlation between macrophage activation and macrophage-hepatocyte communication in the pathogenesis of MASH. Our study first identifies TMEM189 as a critical regulator of the inflammatory response in MDMs during MASH progression.

The well-known function of fatty acid synthase (FASN) is the de novo biosynthesis of fatty acids. FASN condenses acetyl-CoA and malonyl-CoA, converting them into saturated fatty acids through a series of reactions. A key product of the pathway is palmitic acid, a 16-carbon saturated fatty acid that serves as a precursor to long-chain fatty acids. The palmitate produced by the FASN pathway can be reversibly conjugated to the cysteine residues of target proteins in a process of palmitoylation (*S*-acylation) [35]. This post-translational modification enhances protein stability and traffic. Some studies have shown that deletion of *Fasn* in mice reduces LPS- or palmitate-provoked macrophage activation, attenuates atherosclerosis in *ApoE*^–/–^ mice [36], and restrains HFD-induced steatosis and chronic hepatic inflammation [37]. FASN enhances TLR signaling by increasing MYD88 palmitoylation [21], while its palmitoylation of NLRP3 at Cys898 licenses NLRP3 translocation to the Golgi and subsequent inflammasome assembly, thereby promoting NLRP3 inflammasome activation [22]. These studies therefore suggest a role for FASN in inflammasome priming. Our data demonstrate that TMEM189 physically interacts with FASN and negatively regulates its protein expression. In *TMEM189*-deficient macrophages, sustained high levels of FASN promote NLRP3 palmitoylation and NLRP3 inflammasome activation. Conversely, the enhanced NLRP3 inflammasome activation induced by *TMEM189* loss was abolished following pharmacological blockade of FASN or inhibition of palmitoylation, reducing it to a level comparable to that in the control group. This suggests that TMEM189-induced NLRP3 inflammasome activation might occur through FASN-regulated NLRP3 palmitoylation. These findings indicate that TMEM189 might be an endogenous brake on FASN-dependent inflammatory response.

TMEM189/PEDS1-mediated introduction of a vinyl ether double bond endows plasmalogens with special biochemical and biophysical properties, such as antioxidant activity and enhanced membrane fluidity [9, 10]. Additionally, plasmalogens are key components of mammalian cell membranes and lipid rafts, playing a crucial role in maintaining normal membrane structure and function, as well as in intracellular signal transduction [38, 39]. A recent document has indicated that plasmalogens play an important role in myeloid cell biology and inflammation in a zebrafish model [17]. *Tmem189/Peds1*-deficient larvae exhibit reduced total plasmalogen levels, increased IL-1β and NF-κB expression, elevated ROS levels, impaired inflammation resolution, and tissue regeneration. Chronic skin inflammation worsened in *Peds1*-deficient larvae is mitigated by exogenous plasmalogen, which also alleviates hyper-susceptibility to bacterial infection, demonstrating the anti-inflammatory function of *TMEM189/PEDS1-*involved plasmalogen synthesis [17]. Our research on mouse models is similar to that observed in zebrafish. We also observed decreased levels of plasmalogen and increased reactive oxygen species (ROS) in *Tmem189*‑deficient cells (data not shown). Since TMEM189-mediated plasmalogen makes cells more resistant to ROS, thereby protecting the normal physiological function of cells from oxidative stress [40], we did not rule out ROS-induced NLRP3 inflammasome activation in *Tmem189*-deficient macrophages. This may be one of the mechanisms by which TMEM189 regulates the inflammatory response. Additionally, in *Tmem189*-deficient MASH mouse models, although the percentage of KCs was decreased, their absolute number remained unaltered, which may be due to replenishment by MDMs differentiating into KC-like cells. The detailed mechanisms underlying this phenotype require further investigation.

In conclusion, our research identified the protective effect of macrophage-expressed TMEM189 in MASH progression. In diet-induced MASH mice, *Tmem189* loss specifically increased the abundance of MDMs in the liver. Mechanistically, TMEM189 associates with FASN and negatively regulates the inflammatory response by modulating the TMEM189–FASN signaling axis. Our study suggests that TMEM189 is expected to be a potential therapeutic target molecule in metabolic liver diseases, including MASH.

## Materials and methods

### Antibodies and reagents

The antibodies and major reagents used in this study are listed in Supplementary Table 1 and 2.

### *Tmem189* gene conditional knockout (KO)mice

Myeloid-specific *Tmem189* KO mice were generated by crossing *Tmem189*^*fl/fl*^ mice with *Lyz2*^*Cre*^ mice (Cyagen Biosciences Inc., Suzhou, China). Similarly, Kupffer cell-specific *Tmem189* KO mice were generated by crossing *Tmem189*^*fl/fl*^ mice with *Clec4f*^*Cre*^ mice (Cyagen Biosciences Inc., Suzhou, China). All mice used in this study had a C57BL/6 background. The mutant mice exhibited no phenotypic abnormalities or developmental or reproductive defects. All mice were housed in a specific pathogen-free (SPF) facility at a constant room temperature with free access to water and standard mouse chow. All animal experimental procedures and techniques were approved by the Animal Ethics Committee of Peking University Health Sciences Center (LA2022100).

### Mouse models

To establish the STAM model, neonatal mice (postnatal day 2) received a single subcutaneous injection of streptozotocin (200 μg per mouse). Beginning at 4 weeks of age, mice were fed a high-fat diet (HFD; D12492, Research Diets, New Brunswick, NJ) for either 8 weeks to induce steatohepatitis or 14 weeks to induce fibrosis. To establish a diet-induced obesity model, male 8-week mice were maintained on the same HFD for 24 weeks to induce hepatic steatosis. To establish methionine-choline deficient (MCD) model, male 8-week mice were fed an MCD diet (A02082002B, Research Diets, New Brunswick, NJ) for 2 weeks. Age-matched control mice in both models were fed a standard rodent diet.

### AAV8-delivered Tmem189 restoration in hepatic macrophages of *Tmem189*^*fl/fl*^*Lyz2*^*Cre*^ mice

To achieve overexpression of TMEM189 in hepatic macrophages, an adeno-associated virus serotype 8 (AAV8) delivery system was utilized. The AAV8 vectors, which carried either mouse *Tmem189* (*AAV8-Tmem189*) or an empty control (*AAV8-Control*) and targeted the F4/80 macrophage-specific promoter, were manufactured by Tsingke Biotech Co., Ltd. (Beijing, China). *Tmem189*^*fl/fl*^*Lyz2*^*Cre*^ mice received a streptozotocin injection on postnatal day two and were administered recombinant *AAV8-Control* or *AAV8-Tmem189* viral particles (2 × 10^11^/mouse) via tail vein injection at 4 weeks of age. Four weeks post-injection, the mice were fed an HFD for 8 weeks to induce steatohepatitis.

### Plasmid construction

FLAG-FASN plasmids were obtained from Dr. Suowen Xu (University of Science and Technology of China). The GFP-TMEM189, FLAG-TMEM189, GST-TMEM189_1-45_, GST-TMEM189_150-230_, and GST-TMEM189_183-270_ plasmids were constructed in our laboratory [14]. The truncations of FASN were amplified from FLAG-FASN by PCR and then cloned into pGEX-4T-1 to construct GST-FASN_1-428_, GST-FASN_429-817_, GST-FASN_818-1108_, GST-FASN_1109-1405_, GST-FASN_1406-1863_, GST-FASN_1864-2250_ and GST-FASN_2251-2511_ plasmids. Subsequently, GST-TMEM189 and GST-TMEM189_183-230_ plasmids were constructed using similar methods. All the plasmids were confirmed by DNA sequencing.

### Cell isolation, culture and transfection

Bone marrow-derived macrophages (BMDMs) and dendritic cells (BMDCs) were generated as previously described with modifications [41]. Briefly, bone marrow cells were isolated from femurs and tibiae, passed through a 100 μm cell strainer to obtain a single-cell suspension, and differentiated in complete DMEM supplemented with 10% FBS and M-CSF (50 ng/mL) for 7 days, then dissociated using ice-cold TEN buffer (40 mM Tris, 4 mM EDTA, 150 mM NaCl, pH 8.0) and collected by centrifugation (500 × *g*, 5 minutes). The cell pellet was resuspended in complete DMEM for subsequent experiments. BMDCs were obtained by culturing bone marrow cells with M-CSF (50 ng/mL) and IL-4 (1 ng/mL) for 5 days.

For peritoneal macrophages (PMs), mice were intraperitoneally injected with 1 ~ 2 mL of 4% thioglycolate medium 72 hours before sacrifice to induce cell mobilization. PMs were then harvested by peritoneal lavage with ice-cold PBS and cultured in complete DMEM.

Mouse neutrophils were collected from male or female mice intraperitoneally injected with 1 ~ 2 ml of 4% thioglycolate medium 8 ~ 12 hours before being euthanized, and cultured in RPMI 1640 Medium supplemented with 0.5% FBS.

THP-1 cells were cultured in RPMI 1640 Medium supplemented with 10% FBS. HEK293T cells were cultured in complete DMEM supplemented with 10% FBS. Cells were transfected with indicated plasmids using NEOFECT Reagent according to the manufacturer’s instructions.

### Generation of stable *TMEM189* knockdown cell lines

To establish stable *TMEM189* knockdown in THP-1 cells, we employed a lentiviral-mediated RNA interference strategy. The lentiviral plasmid *pLVX-shRNA2-Puro-TMEM189* was constructed by cloning shRNA targeting the *TMEM189* sequence (5’-GCTCAACTACCCTCTGGAGAA-3’) into the pLVX-shRNA2-Puro backbone. High-titer *pLXV-shRNA TMEM189* (*shTMEM189*) or *pLXV-shRNA control* (*shcontrol*) lentiviral particles were packaged into HEK293T cells. After infection of THP-1 cells, stable cells containing *shTMEM189* or *shcontrol* were selected using puromycin. The knockdown efficiency was confirmed by both qRT-PCR and western blotting analysis.

### Co-Immunoprecipitation (Co-IP) assay and western blotting

For co-immunoprecipitation (co-IP) analysis, the treated cells were collected and lysed in IP lysis buffer supplemented with protease inhibitors (Roche Diagnostics, 04693116001) and phosphatase inhibitors. Total cell lysates were precleared by incubation with Protein G Sepharose™ Fast Flow for 30 min at 4 °C to minimize nonspecific binding. Following this, the precleared lysates were incubated overnight at 4 °C with either Anti-GFP Affinity Beads (Smart Lifesciences, SA070001) or DYKDDDDK Tag (FLAG) Nanoselector Agarose (Dakewe Biotech Co., Ltd., 016-101-002). The beads were collected by centrifugation, washed thrice, and resuspended in 2 × loading buffer. The immunoprecipitated complexes were eluted and analyzed by western blotting.

For western blotting, protein concentrations were determined using the BCA Protein Assay Kit (Beyotime, P0010). Equal amounts of protein were separated by SDS-PAGE and then transferred to nitrocellulose membranes (Millipore, HATF00010). After blocking with 5% skimmed milk, the membranes were incubated sequentially with the corresponding primary and secondary antibodies. After washing, the protein bands were visualized using an enhanced chemiluminescence substrate and imaged with a chemiluminescent imaging system (iBright 750, ThermoFisher Scientific). Band intensities were quantified using the ImageJ software. All data are representative of at least three independent experiments.

### In vitro GST-pulldown assay

Soluble recombinant GST, GST-TMEM189, GST-TMEM189 mutants, and GST-FASN mutants were expressed in *E. coli* and immobilized onto glutathione-Sepharose 4B beads. The beads were incubated with whole-cell lysates extracted from treated HEK293T or THP-1 cells overnight at 4 °C. After extensive washing, the beads were resuspended in 2 × SDS loading buffer, and the bound proteins were eluted and analyzed by Western blotting.

### Acyl-biotin exchange (ABE) Assay

The ABE assay was carried out with the IP-ABE Palmitoylation Kit following the manufacturer’s protocol. In brief, protein palmitoylation was assessed using the Acyl-Resin-Assisted Capture (Acyl-RAC) approach, which involves blocking, hydroxylamine cleavage, labelling, elution, and detection. After immunoprecipitation of target proteins from cell lysates with anti-NLRP3 antibody‑coupled beads overnight at 4 °C, the beads were incubated with N‑ethylmaleimide (NEM) for 30 min at room temperature to block unmodified cysteine residues. Following extensive washes, the beads were divided equally into two sets: the +HAM group was treated with hydroxylamine (HAM) for 1 hour at room temperature to specifically cleave palmitoyl‑thioester linkages, whereas the –HAM control group received HAM‑free buffer. Newly exposed thiol groups resulting from palmitoylation in the +HAM samples were then labelled with thiol‑reactive biotin‑HPDP for 1 hour at room temperature. After repeated washing steps, proteins were eluted and analysed by immunoblotting using an anti‑biotin antibody for detection.

### RNA extraction and quantitative real-time PCR (qRT-PCR) assays

Total RNA was extracted from indicated samples using TRIzol reagent (Invitrogen, 15596026CN). Subsequently, 1 μg of RNA was reverse-transcribed into cDNA using the RevertAid First Strand cDNA Synthesis Kit (ThermoFisher Scientific, K1622) according to the manufacturer’s protocol. qRT-PCR analysis was performed using SYBR Green qPCR Mix (Vazyme, Q712-03). The primers against the indicated genes used in this study were listed in Table S3, and β-actin (ACTB) served as the endogenous control for normalization.

### Serum chemistry measurements

Mouse serum was collected from blood and assayed for alanine aminotransferase (ALT), aspartate aminotransferase (AST), triglyceride (TG), and total cholesterol (TC) using commercial diagnostic kits (Applygen, Beijing, China) according to the manufacturer’s instructions.

### Enzyme-linked immunosorbent assay (ELISA)

ELISA kits for human and mouse TNF-α, IL-6, IL-1β, or mouse MCP-1 were purchased from Proteintech Group (Wuhan, China). Different cell culture supernatants were collected and subjected to ELISA assays according to the manufacturer’s instructions.

### Isolation of mouse primary hepatocytes

Mice were euthanized by CO_2_ asphyxiation. Mouse primary hepatocytes were isolated using a two-step collagenase perfusion protocol [42]. In brief, the liver was first perfused with 1 × Hank’s buffered salt solution (HBSS; without magnesium or calcium) containing 0.5 mmol/L EDTA, followed by perfusion with DMEM-low glucose supplemented with 15 mmol/L HEPES and 0.03% collagenase IV type (179.4 U/mL, Sigma). The digested liver was then excised, transferred to a Petri dish, and gently minced in Williams’E medium. The resulting cell suspension was filtered through a 100 μm mesh and subsequently centrifuged at 40 × g for 3 minutes at 4 °C. The pellet was washed three times with cold Williams’E Medium. Finally, the hepatocytes were resuspended in Williams’E medium supplemented with 10% fetal bovine serum (FBS), 2 mmol/L L-glutamine, and 1 mol/L insulin, and then plated on Type I collagen-coated plates. Cells were maintained at 37 °C in a humidified incubator with 5% CO_2_.

### Isolation of hepatic macrophages

Mice were euthanized by CO_2_ asphyxiation. Mouse hepatic macrophages were isolated following the established protocol described in a previous study [43, 44]. Briefly, the liver was perfused using the two-step collagenase protocol mentioned above. Dissociated liver cells were passed through a 100 μm mesh and then centrifuged at 40 × *g* for 3 minutes to remove hepatocytes. The resulting supernatant was centrifuged at 800 × *g* for 7 minutes. After discarding the supernatant, the pellet containing non-parenchymal cells was resuspended. The non-parenchymal cell suspension was then layered onto a discontinuous isotonic gradient and centrifuged at 800 × *g* for 15 minutes. Hepatic macrophages were collected from the 25% Percoll layer and centrifuged at 1350 × *g* for 15 minutes. After the cells were extracted from the middle layer and centrifuged, the resulting pellet was resuspended. The cells were then further purified by flow cytometric sorting for the CD45^+^Ly6G^-^CD11b^+^F4/80^+^. Finally, the isolated hepatic macrophages were collected and further analyzed by flow cytometry, western blotting, or quantitative real-time PCR (qRT-PCR).

### Flow cytometry analysis

Mice were euthanized by CO_2_ asphyxiation. Single-cell suspensions were prepared from peritoneal lavage, peripheral blood, bone marrow, spleen, and liver from different mice. The cells were then stained with different fluorescein-labeled antibodies at 4 °C for 30 minutes and analyzed by flow cytometry using either a FACS Verse or Canto system (BD Biosciences).

Mouse liver monocytes and neutrophils were separated by collagenase IV, DNase I, and Percoll. Different cells were stained with fluorescein-labeled antibodies and gated according to a previously described strategy [45].

### Histopathological and immunohistochemical analysis

Mouse livers were sectioned and fixed overnight in 4% paraformaldehyde, dehydrated through a graded ethanol series, embedded in paraffin, and sliced into 4 μm sections. To evaluate hepatic steatosis and fibrosis, sections were stained with hematoxylin and eosin (H&E) and Sirius red according to standard protocols. Additionally, portions of liver tissue were embedded in OCT compound and frozen for cryosectioning. The resulting frozen sections were stained with Oil Red O (ORO) to visualize lipid deposition.

For immunohistochemistry staining, paraffin-embedded sections were deparaffinized, rehydrated, and subjected to antigen retrieval in citrate buffer (pH 6.0). After blocking with 10% goat serum for 30 minutes, the sections were incubated overnight at 4 °C with a primary antibody against F4/80 (Servicebio, GB113373), followed by a 1-hour incubation with a secondary antibody. Signal was developed using DAB (3,3’-Diaminobenzidine hydrochloride, GK600705), and images were captured using a microscope (Olympus, BX-UCB).

### Immunofluorescence and confocal microscopy analysis

For tyramide signal amplification (TSA)-based multispectral immunofluorescence, a sequential staining protocol was employed. In the first round, sections were incubated with an anti-F4/80 antibody overnight at 4 °C, followed by incubation with an HRP-polymer-conjugated goat anti-mouse secondary antibody and subsequent development with HyperFluorTM 488 TSA (ApexBio, K4301; 1:100) for 10 minutes. Following antigen retrieval and blocking, the second round of staining was performed using an anti-TMEM189 antibody (Sigma Aldrich, T8953; 1:500), an HRP-polymer-conjugated goat anti-rabbit secondary antibody and Cy3 TSA (ApexBio, K1051; 1:100). Finally, nuclei were stained with Hoechst 33342 for 10 minutes, sections were mounted with fluorescence Mounting Medium (Solarbio, S2100), and images were acquired using a confocal microscope (LSM 880 Meta plus Zeiss Axiovert zoom, Zeiss).

### Statistical analysis

All data are presented as mean ± standard deviation (SD). Differences between two groups were compared using Student’s t-test (two-tailed, unpaired). Differences between multiple groups were compared using one-way ANOVA followed by Tukey’s honestly significant difference (HSD) post hoc test. All statistical analyses were performed using GraphPad Prism 9.0 (GraphPad Software, San La Jolla, CA, USA). Statistical significance is indicated as *p < 0.05, **p < 0.01, ***p < 0.001; ns, not significant.

## Supplementary information

Supplementary Figures and Figure Legends

Supplementary Table 1

Supplementary Table 2

Supplementary Table 3

## Data Availability

The data can be made available upon reasonable request to the corresponding author at the following address: Yingyu Chen, Department of Immunology, Peking University School of Basic Medical Sciences, 38 Xueyuan Road, Beijing, 100191, China. Email: yingyu_chen@bjmu.edu.cn.
