# Supplementary material for: TMEM189/PEDS1 deficiency aggravates MASH progression by promoting FASN-mediated macrophage activation
Source: Cell Death Dis. 2026 Aug 13;17(1):956. doi: 10.1038/s41419-026-09145-7 (PMC13748651; doi:10.1038/s41419-026-09145-7)
Supplement: Supplementary file 1 [file 41419_2026_9145_MOESM1_ESM.pdf]

Supplementary Materials

TMEM189/PEDS1 deficiency aggravates MASH progression by promoting FASN-mediated macrophage activation

Pengli Guo[1], Jinqiu Feng[2], Yaxin Lou[3], Tao Li[1], Shufang Ye[1], Yongjun Zhang[1], Chunlei Wang[1], Ping Lv[1], Zongming Zhang[2], Yingyu Chen[1,4,*]

[1]Department of Immunology, Peking University School of Basic Medical Sciences, NHC Key Laboratory of Medical Immunology, Peking University, Beijing 100191, China;

[2]Department of General Surgery, Beijing Electric Power Hospital, State Grid Corporation of China; Clinical Medical Research Center for Hepatobiliary Diseases in General Surgery, China General Technology Group, Beijing 100073, China.

[3]Medical and Healthy Analytical Center, Peking University, Beijing 100191, China;

[4]Center for Human Disease Genomics, Peking University, Beijing 100191, China.

*Corresponding authors. Email addresses: zhangzongming@mail.tsinghua.edu.cn or yingyu_chen@bjmu.edu.cn.

Supplemental Figures and Figure Legends

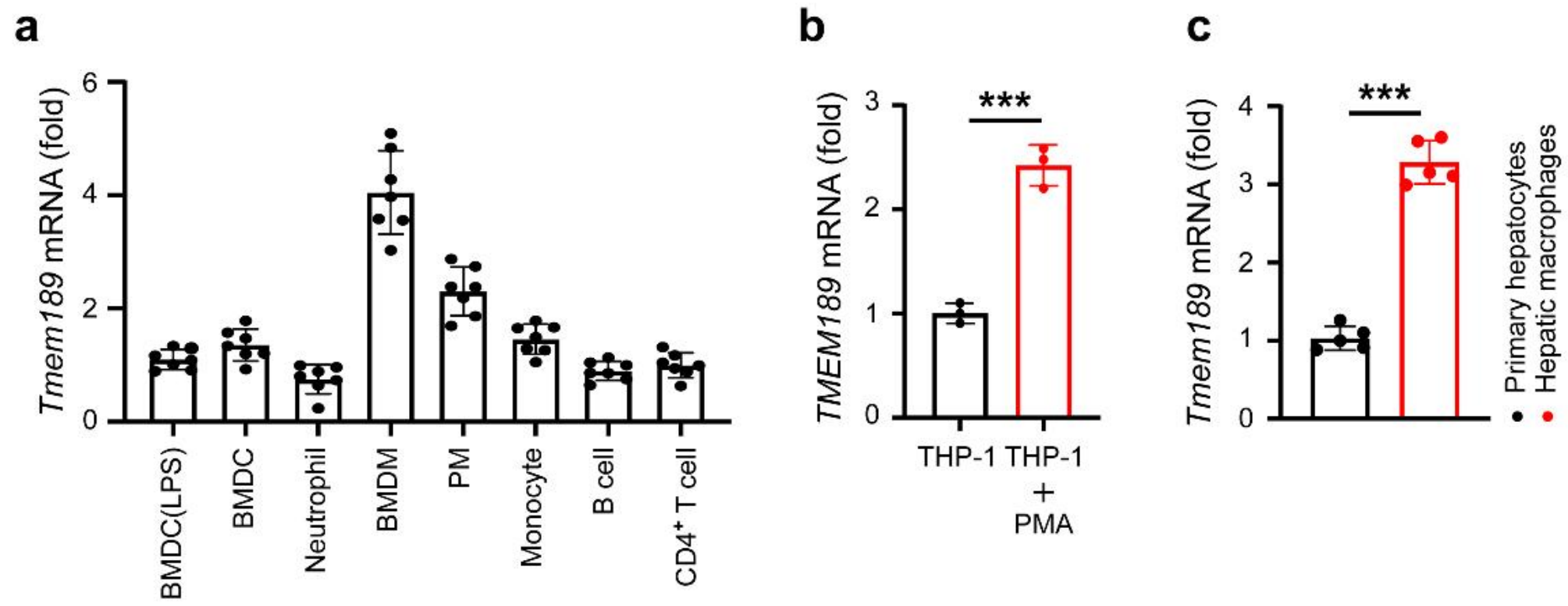

Fig. S1 TMEM189 is highly expressed in macrophages. (**a**) qRT-PCR analysis of *Tmem189* mRNA levels in various mouse immune cells (n = 7). (**b**) *TMEM189* mRNA levels in THP-1 or PMA-differentiated THP-1 cells were measured by qRT-PCR. (**c**) qRT-PCR analysis of *Tmem189* mRNA levels in primary hepatocytes and hepatic macrophages from wild-type mice (n = 5). Statistical significance was determined using the unpaired two-tailed Student's *t*-test (b, c). Data are presented as mean ± SD. ***p <0.001.

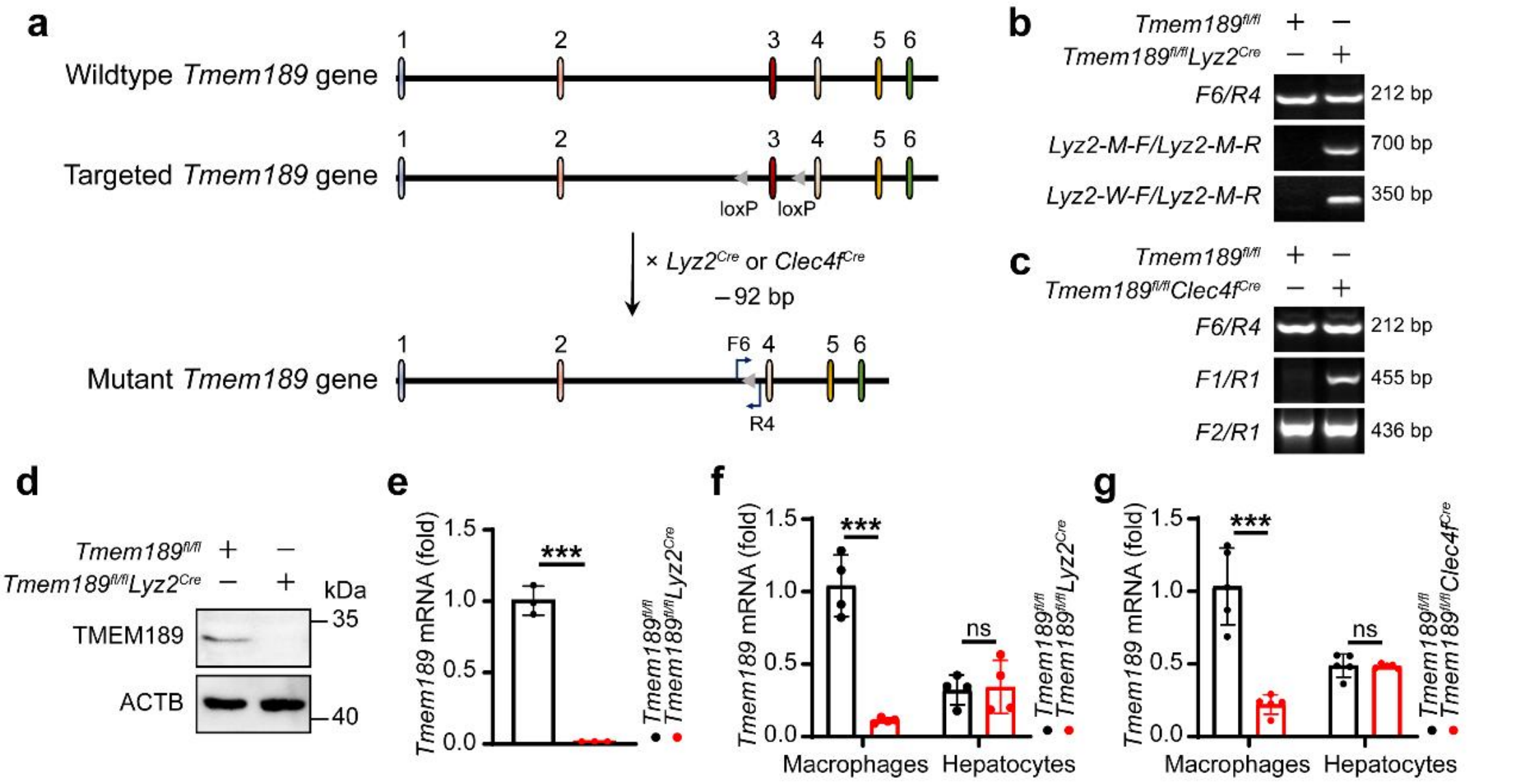

Fig. S2 Generation and validation of myeloid-specific or Kupffer cell-specific *Tmem189* knockout mice. (**a**) The genomic structure of wildtype and mutant *Tmem189* gene is shown. Homozygous *Tmem189*-floxed mice were crossed with *Lyz2*^{Cre} or *Clec4f*^{Cre} transgenic mice in several breeding steps to ultimately produce *Tmem189*$^{fl/fl}$*Lyz2*^{Cre} or *Tmem189*$^{fl/fl}$*Clec4f*^{Cre} mice, respectively. (**b** and **c**) Genomic PCR was used to identify the mutations of *Tmem189* gene in different mice. (**d** and **e**) BMDMs were isolated from *Tmem189*$^{fl/fl}$ (*Tmem189 WT*) or *Tmem189*$^{fl/fl}$*Lyz2*^{Cre} (*Tmem189 KO*) mice. The levels of Tmem189 were analyzed by western blotting and qRT-PCR. (**f** and **g**) Hepatic macrophages ($CD45^+CD11b^+Ly6G^-F4/80^+$) and primary hepatocytes were isolated from *Tmem189*$^{fl/fl}$ or *Tmem189*$^{fl/fl}$*Lyz2*^{Cre} (f) or *Tmem189*$^{fl/fl}$*Clec4f*^{Cre} (g) mice, and *Tmem189* mRNA levels were measured by qRT-PCR (n = 4). Statistical significance was determined using the unpaired two-tailed Student's *t*-test (e) and multiple *t*-test with the two-stage linear step-up procedure of Benjamini, Krieger and Yekutieli (f, g). Data are presented as mean ± SD. ns, not significant, ***p <0.001.

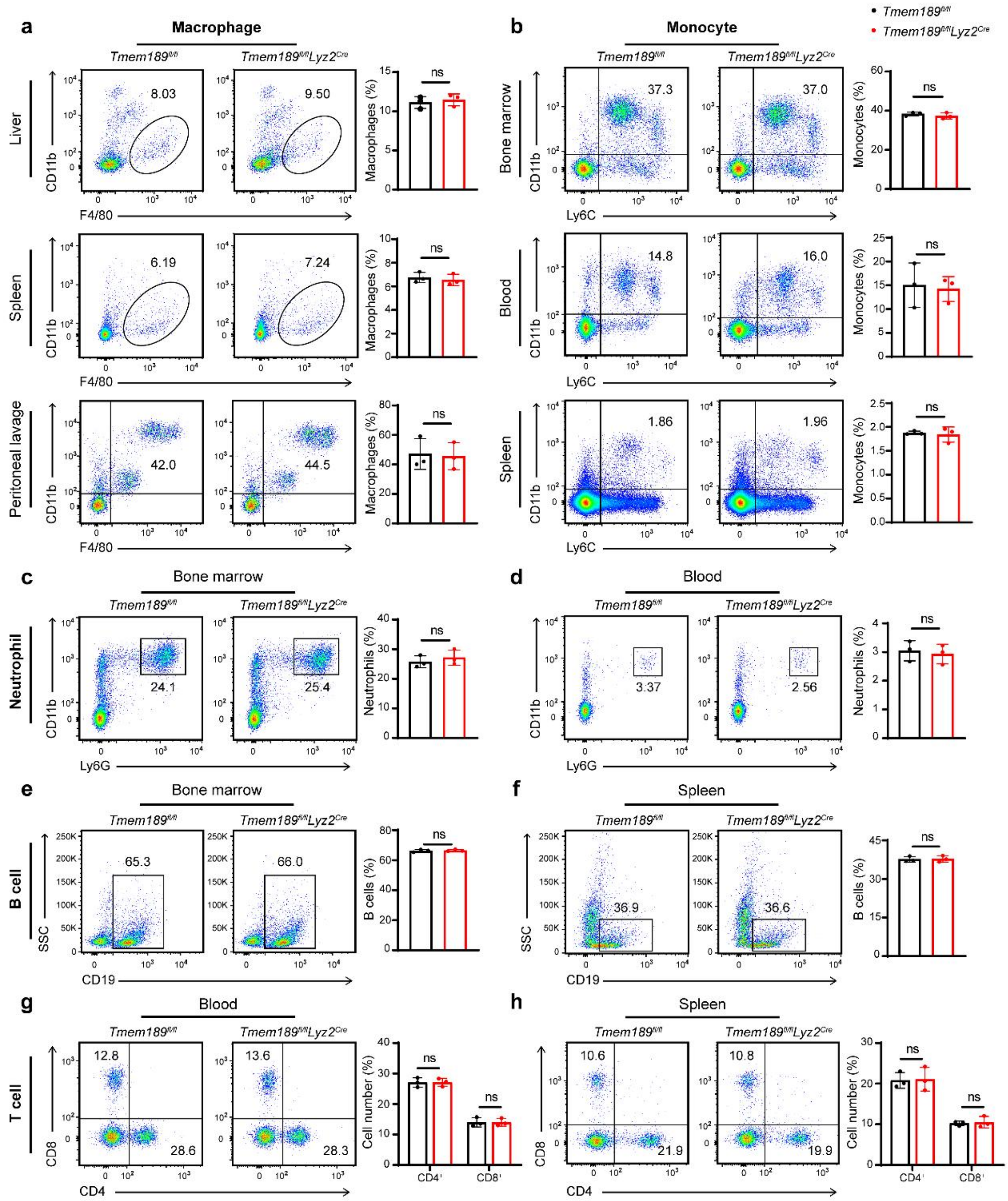

Fig. S3 *Tmem189* deficiency fails to affect the development of immunocytes. The percentage of $CD45^+CD11b^+F4/80^+$ macrophages (**a**), $CD45^+CD11b^+Ly6C^+$ monocytes (**b**), $CD45^+CD11b^+Ly6G^+$ neutrophils (**c** and **d**), $CD45^+CD3^+CD19^+$ B cells (**e** and **f**), $CD45^+CD3^+CD4^+$ or $CD45^+CD3^+CD8^+$ T cells (**g** and **h**) in indicated tissues from *Tmem189*$^{fl/fl}$ or *Tmem189*$^{fl/fl}$*Lyz2*^{Cre} mice were analyzed by flow cytometry and quantified (n = 3). Statistical significance was determined using the multiple *t*-test with the two-stage linear step-up procedure of Benjamini, Krieger and Yekutieli (a-h). Data are presented as mean ± SD. ns, not significant.

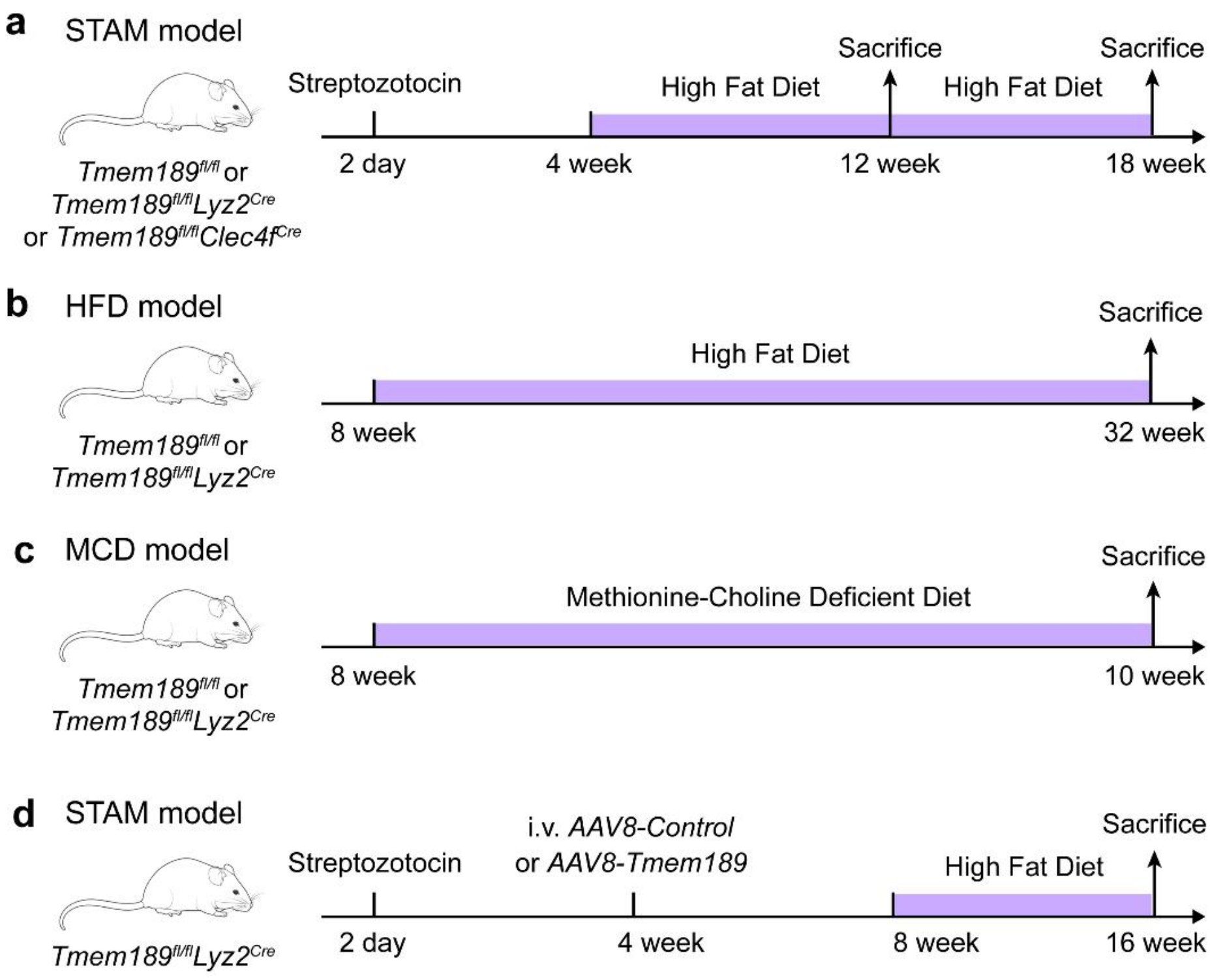

Fig. S4 Schematic illustration of the establish of mouse models. (**a**) STAM model: *Tmem189*$^{fl/fl}$, *Tmem189*$^{fl/fl}$*Lyz2*^{Cre} or *Tmem189*$^{fl/fl}$*Clec4f*^{Cre} male mice received a streptozotocin injection on postnatal day two and were placed on a high-fat diet (HFD) starting at 4 weeks of age. After 8 or 16 weeks, these mice were euthanized. (**b**) HFD model: *Tmem189*$^{fl/fl}$ or *Tmem189*$^{fl/fl}$*Lyz2*^{Cre} male mice were fed an HFD beginning at 8 weeks of age, and were sacrificed 24 weeks later. (**c**) MCD model: *Tmem189*$^{fl/fl}$ or *Tmem189*$^{fl/fl}$*Lyz2*^{Cre} male mice were fed an MCD diet beginning at 8 weeks of age, and were euthanized 2 weeks later. (**d**) *Tmem189*$^{fl/fl}$*Lyz2*^{Cre} male mice received a streptozotocin injection on postnatal day two and administered with recombinant *AAV8-Vectors* (*AAV8-Control* or *AAV8-Tmem189* carrying macrophage-specific F4/80 promoter) via tail vein injection at a dose of 2×10^{11} viral particles per mouse at 4 weeks of age. Four weeks post-injection, these mice were placed on an HFD for 8 weeks and then euthanized.

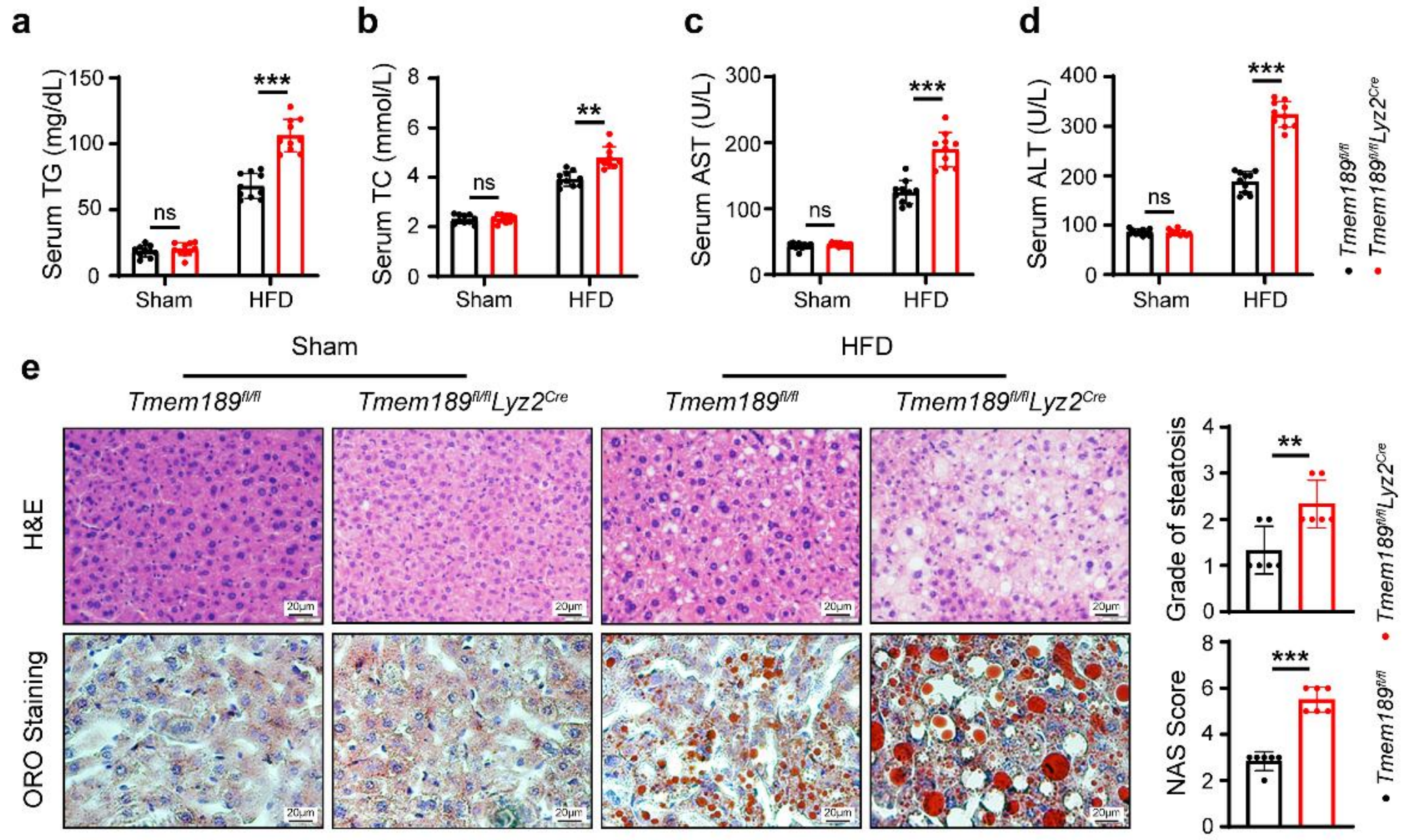

Fig. S5 *Tmem189* knockout in myeloid cells aggravates HFD-induced MASH. (**a-d**) Serum levels of TG (**a**), TC (**b**), AST (**c**) and ALT (**d**) were measured in different mice (n = 10). (**e**) Representative images of liver tissues stained with H&E or ORO from different groups. The grade of steatosis and NAS score per field were quantified, respectively. Statistical significance was determined using the multiple *t*-test with the two-stage linear step-up procedure of Benjamini, Krieger and Yekutieli (a-d), unpaired two-tailed Student's *t*-test (e). Data are presented as mean ± SD. ns, not significant, $^{**}p$ <0.01, $^{***}p$ <0.001.

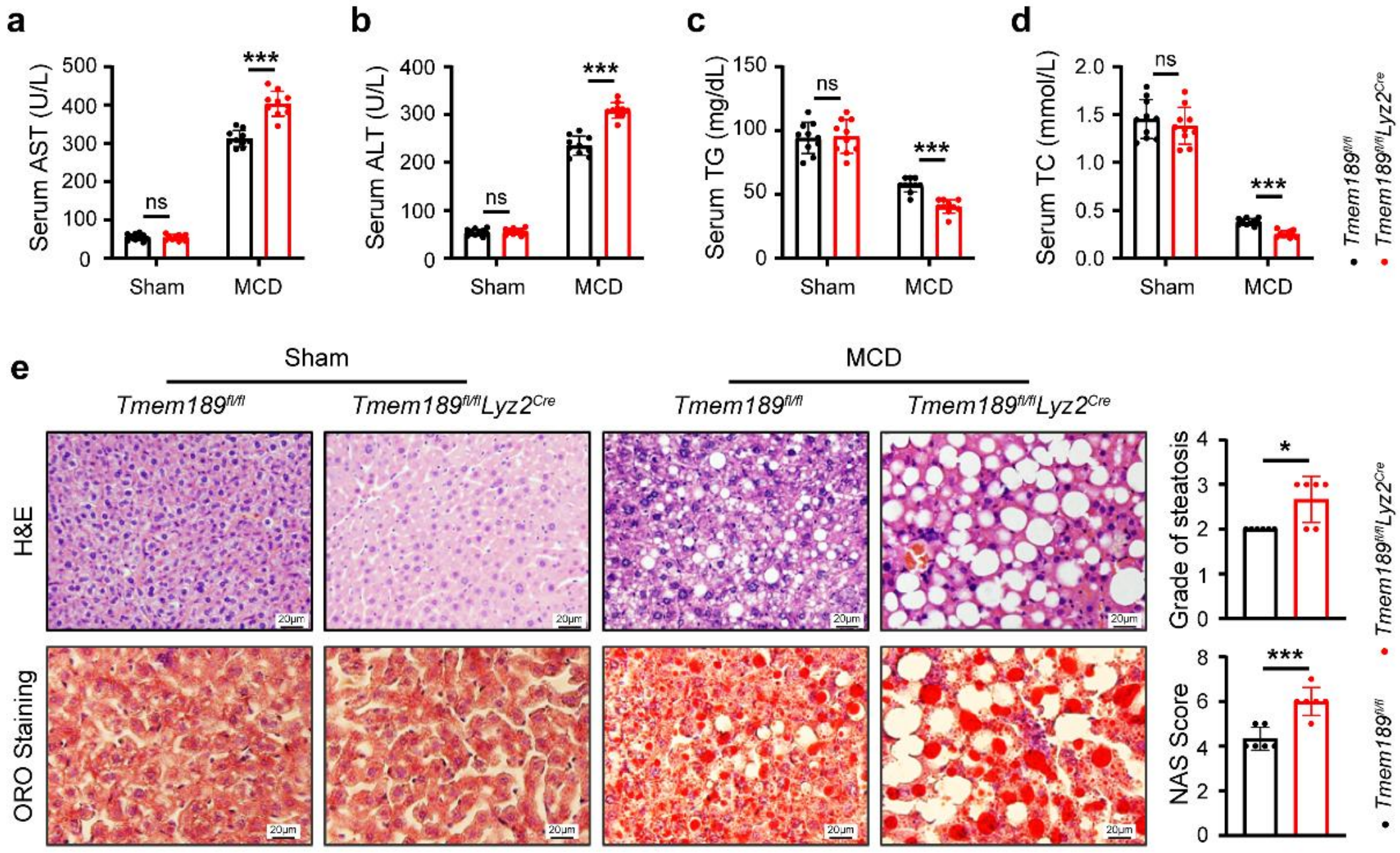

Fig. S6 *Tmem189* deletion in myeloid cells exacerbates MCD-induced MASH. (**a-d**) Serum levels of AST (**a**), ALT (**b**), TG (**c**) and TC (**d**) were measured in different mice (n = 10). (**e**) Representative images of liver tissues stained with H&E or ORO from different groups. The grade of steatosis and NAS score per field were quantified, respectively. Statistical significance was determined using the multiple *t*-test with the two-stage linear step-up procedure of Benjamini, Krieger and Yekutieli (a-d), unpaired two-tailed Student's *t*-test (e). Data are presented as mean ± SD. ns, not significant, *p <0.05, ***p <0.001.

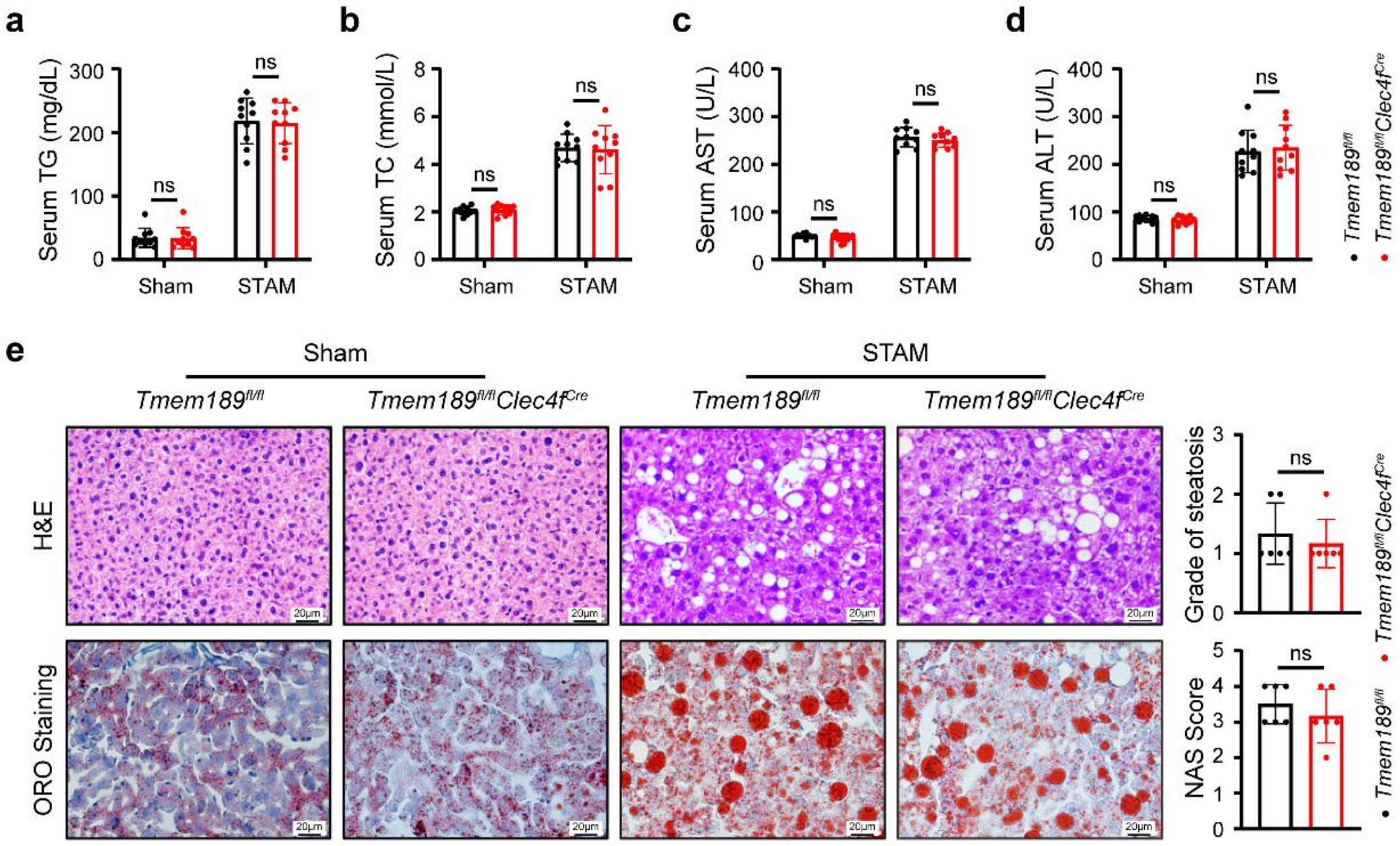

Fig. S7 *Tmem189* loss in Kupffer cells has no determinate effect on diet-induced MASH progression. (**a-d**) Serum levels of TG (**a**), TC (**b**), AST (**c**) and ALT (**d**) were measured in different mice (n = 10). (**e**) Representative images of liver tissues stained with H&E or ORO from different groups. The grade of steatosis and NAS score per field were quantified, respectively. Statistical significance was determined using the multiple *t*-test with the two-stage linear step-up procedure of Benjamini, Krieger and Yekutieli (a-d), unpaired two-tailed Student's *t*-test (e). Data are presented as mean ± SD. ns, not significant.

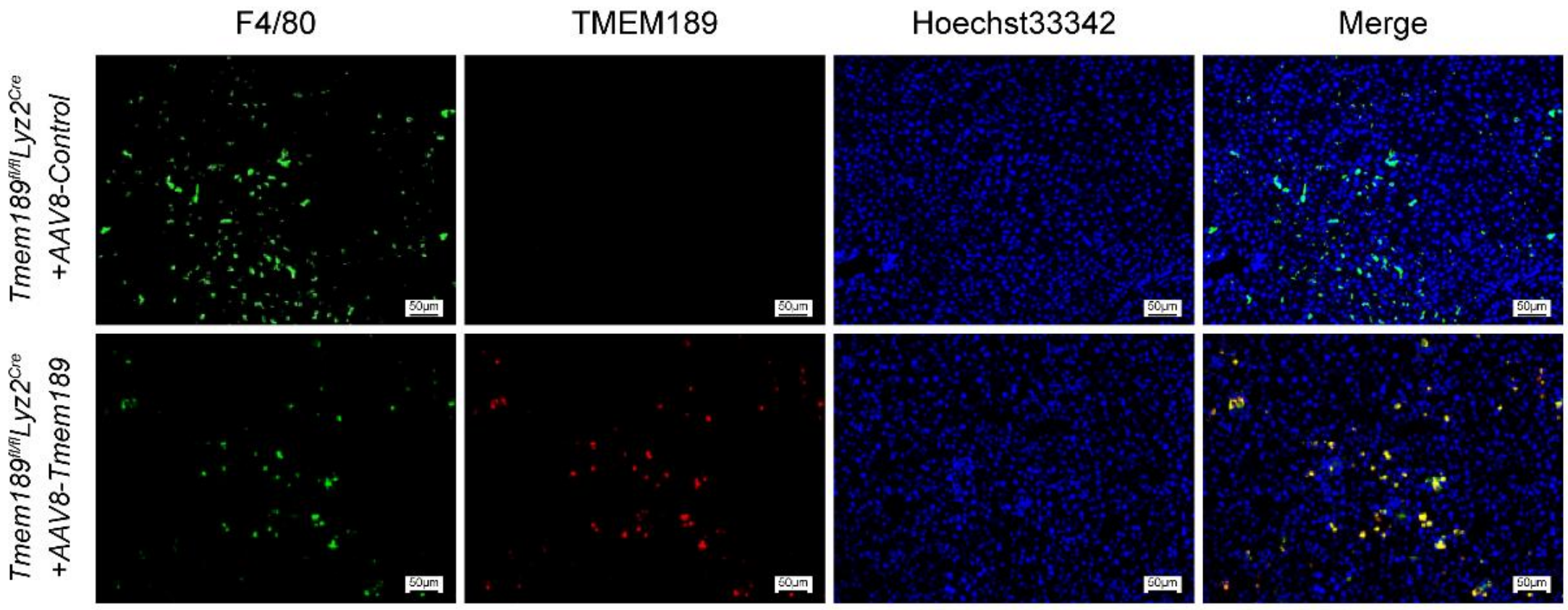

Fig. S8. Overexpression of Tmem189 protein in hepatic macrophages in *Tmem189$^{fl/fl}$Lyz2^{Cre}* mice. *Tmem189$^{fl/fl}$Lyz2^{Cre}* male mice received a streptozotocin injection on postnatal day two and administered with recombinant *AAV8-Vectors* (*AAV8-Control* or *AAV8-Tmem189* carrying macrophage-specific F4/80 promoter) via tail vein injection at 4 weeks of age. Four weeks post- injection, these mice were placed on an HFD for 8 weeks and then euthanized. Representative image of Tmem189 protein and F4/80 immunofluorescence staining in mouse livers were visualized by Confocal microscope. Scale bar, 100 μm.

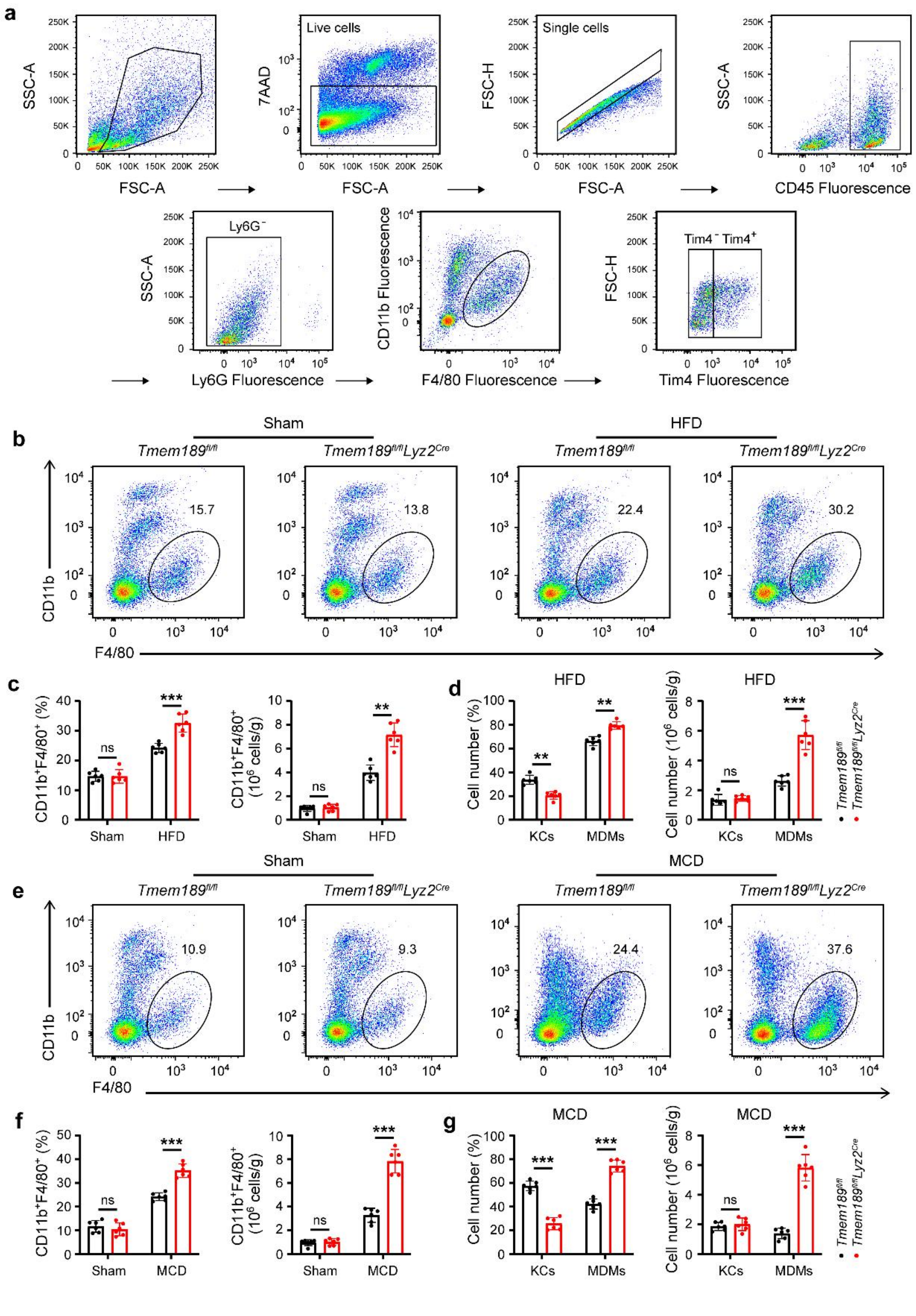

Fig. S9 *Tmem189* KO promotes hepatic macrophage infiltration by preferentially increasing monocyte-derived macrophages (MDMs) in diet-induced MASH mice. (**a**) Flow cytometry gating strategy for analysis of hepatic macrophages ($CD45^+Ly6G^-CD11b^+F4/80^+$), KCs ($CD45^+Ly6G^-CD11b^+F4/80^+Tim4^+$), and MDMs ($CD45^+Ly6G^-CD11b^+F4/80^+Tim4^-$). (**b**) Representative images of flow cytometry

analysis of hepatic macrophages ($CD45^{+}Ly6G^{-}CD11b^{+}F4/80^{+}$) from indicated HFD mice. (**c**) The percentage and total number of hepatic macrophages were quantified by flow cytometry analysis in indicated mice (n = 6). (**d**) The percentage and total number of MDMs ($CD45^{+}Ly6G^{-}CD11b^{+}F4/80^{+}Tim4^{-}$) or KCs ($CD45^{+}Ly6G^{-}CD11b^{+}F4/80^{+}Tim4^{+}$) in the indicated mice were detected by flow cytometry (n = 6). (**e**) Representative images of flow cytometry analysis to hepatic macrophages ($CD45^{+}Ly6G^{-}CD11b^{+}F4/80^{+}$) from indicated MCD mice. (**f**) The percentage and total number of hepatic macrophages were quantified by flow cytometry analysis (n = 6). (**g**) The percentage and total number of MDMs or KCs in the indicated mice were detected by flow cytometry (n = 6). Statistical significance was determined using the multiple *t*-test with the two-stage linear step-up procedure of Benjamini, Krieger and Yekutieli (c, d, f, g). Data are presented as mean ± SD. ns, not significant, **p <0.01, ***p <0.001.

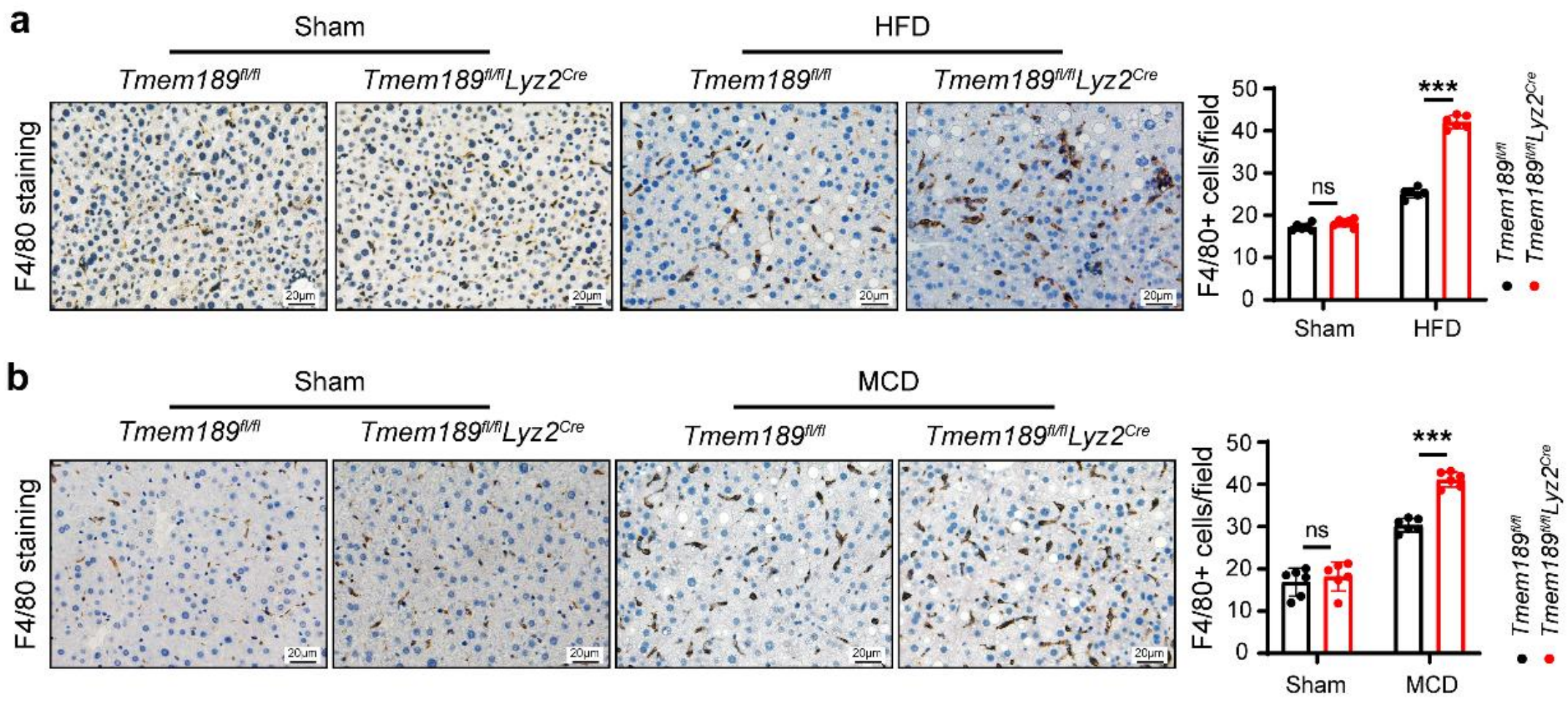

Fig. S10 *Tmem189* depletion increases F4/80^{+} macrophages abundance in MASH mouse livers. (**a** and **b**) Representative images of F4/80 immunohistochemistry staining in different mouse livers (left). The number of F4/80^{+} cells per field were quantified (right). Statistical significance was determined using the multiple *t*-test with the two-stage linear step-up procedure of Benjamini, Krieger and Yekutieli (a, b). Data are presented as mean ± SD. ns, not significant, ***p <0.001.

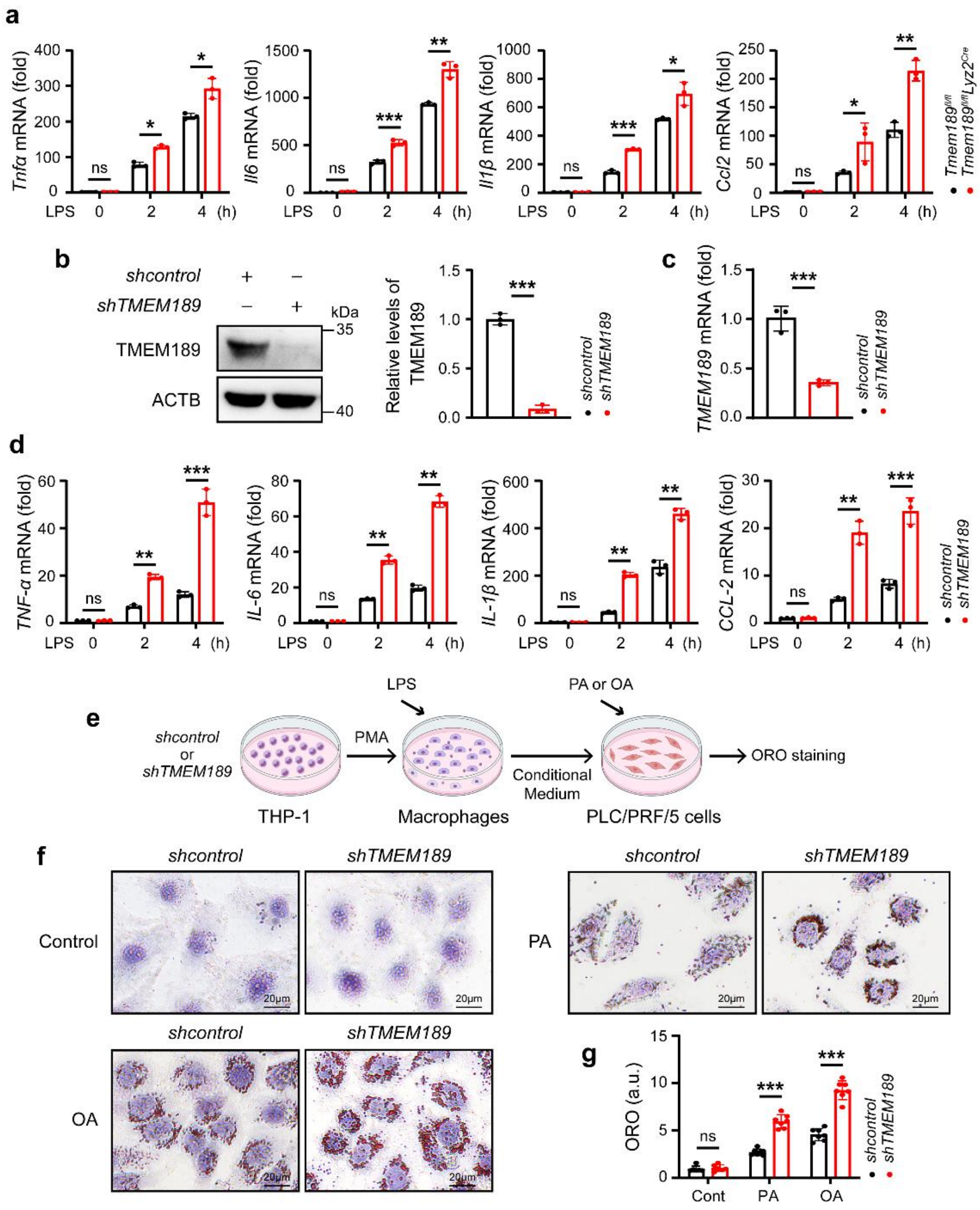

Fig. S11 Loss of *TMEM189* promotes the activation of macrophages and exacerbates hepatocytes steatosis. (**a**) BMDMs from *Tmem189*$^{fl/fl}$ or *Tmem189*$^{fl/fl}$*Lyz2*^{Cre} mice were stimulated with LPS (100 ng/mL) for indicated time. The levels of *Tnfα*, *Il6*, *Il1β* and *Ccl2* mRNA were measured by qRT-PCR. (**b**) The levels of TMEM189 in PMA-differentiated *shcontrol* or *shTMEM189* THP-1 cells were detected by western blotting. The amounts of TMEM189 relative to ACTB were quantified, with the average value in *shcontrol* normalized to 1. (**c**) The mRNA levels of *TMEM189* in PMA-differentiated *shcontrol* or *shTMEM189* THP-1 cells were analyzed by qRT-PCR (**d**) PMA-differentiated *shcontrol* or *shTMEM189* THP-1 cells

were treated with LPS (100 ng/mL) for the indicated times. qRT-PCR measured the mRNA levels of *TNF-α*, *IL-6*, *IL-1β*, and *CCL-2*. (**e**) Schematic illustration of the experimental procedure. PMA-differentiated THP-1 cells containing *shcontrol* or *shTMEM189* were stimulated with LPS (1 μg/mL) for 24 hours. The resulting conditioned medium was collected and co-cultured with PLC/PRF/5 cells for 12 hours, then treated with PA (0.2 mM) or OA (0.1 mM) for 8 hours, and analyzed by ORO staining. (**f**) Representative images of ORO staining in PLC/PRF/5 cells. (**g**) Quantitative analysis of ORO staining by Image J software. Statistical significance was determined using the multiple *t*-test with the two-stage linear step-up procedure of Benjamini, Krieger and Yekutieli (a, d, g) and unpaired two-tailed Student's *t*-test (b, c). Data are presented as mean ± SD. ns, not significant, *$p < 0.05$, **$p < 0.01$, ***$p < 0.001$.

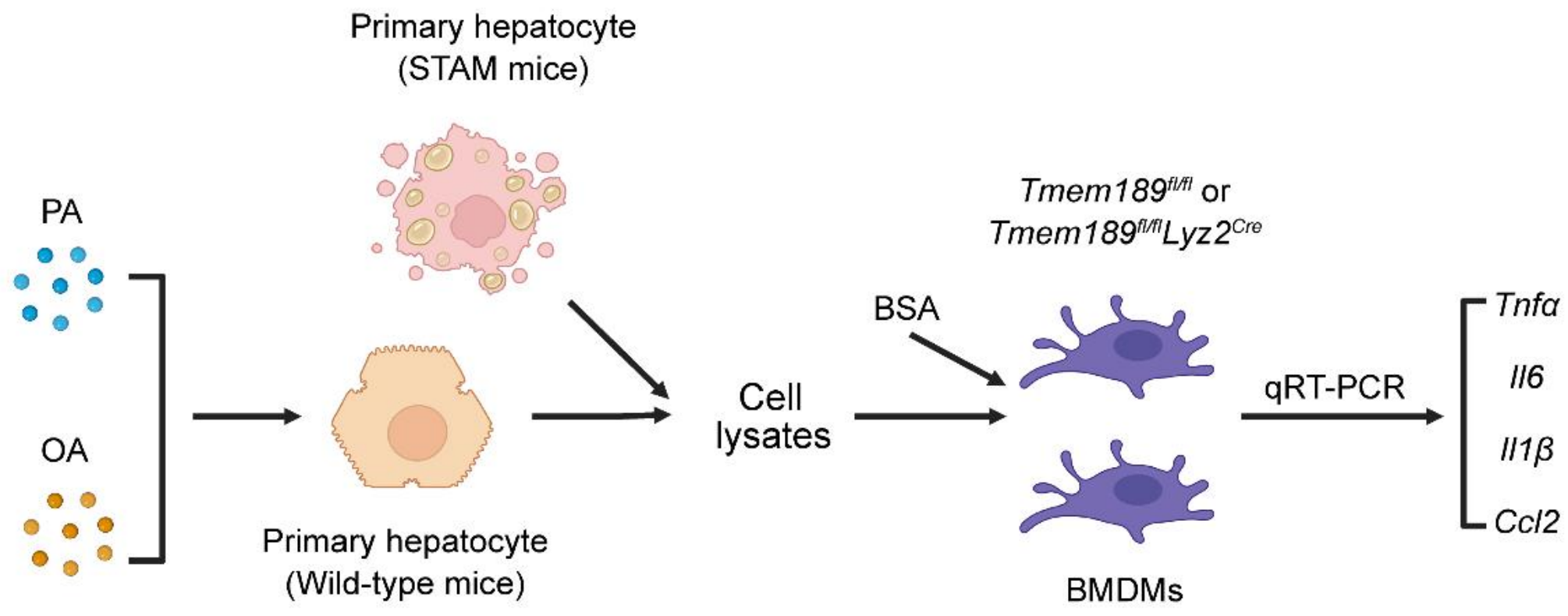

Fig. S12 Schematic illustration of the experimental procedure. Primary hepatocytes were isolated from STAM mice as well as from normal wild-type mice. The latter hepatocytes were treated with palmitic acid (PA, 0.4 mM) or oleic acid (OA, 0.2 mM) for 8 hours. Cell lysates were then prepared by subjecting the hepatocytes to three freeze-thaw cycles, followed by centrifugation to collect the supernatant. These lysates were subsequently co-cultured with BMDMs obtained from *Tmem189*$^{fl/fl}$ and *Tmem189*$^{fl/fl}$*Lyz2*^{Cre} mice for 6 hours. The levels of specific inflammatory factors were assessed using qRT-PCR.

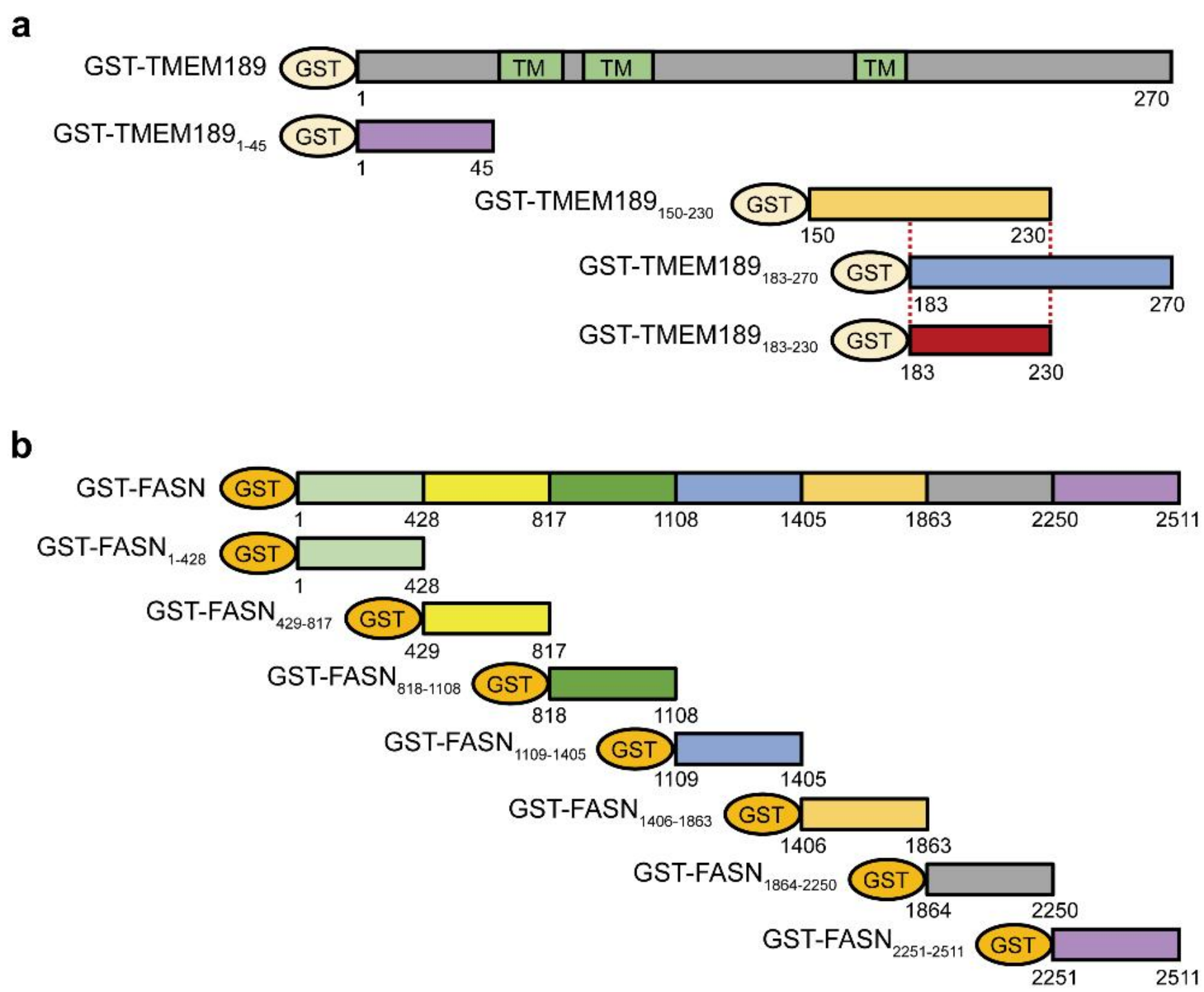

Fig. S13 Schematic illustration of truncation construction. (**a**) Truncations of GST-tagged TMEM189. (**b**) Truncations of GST-tagged FASN fusion protein.

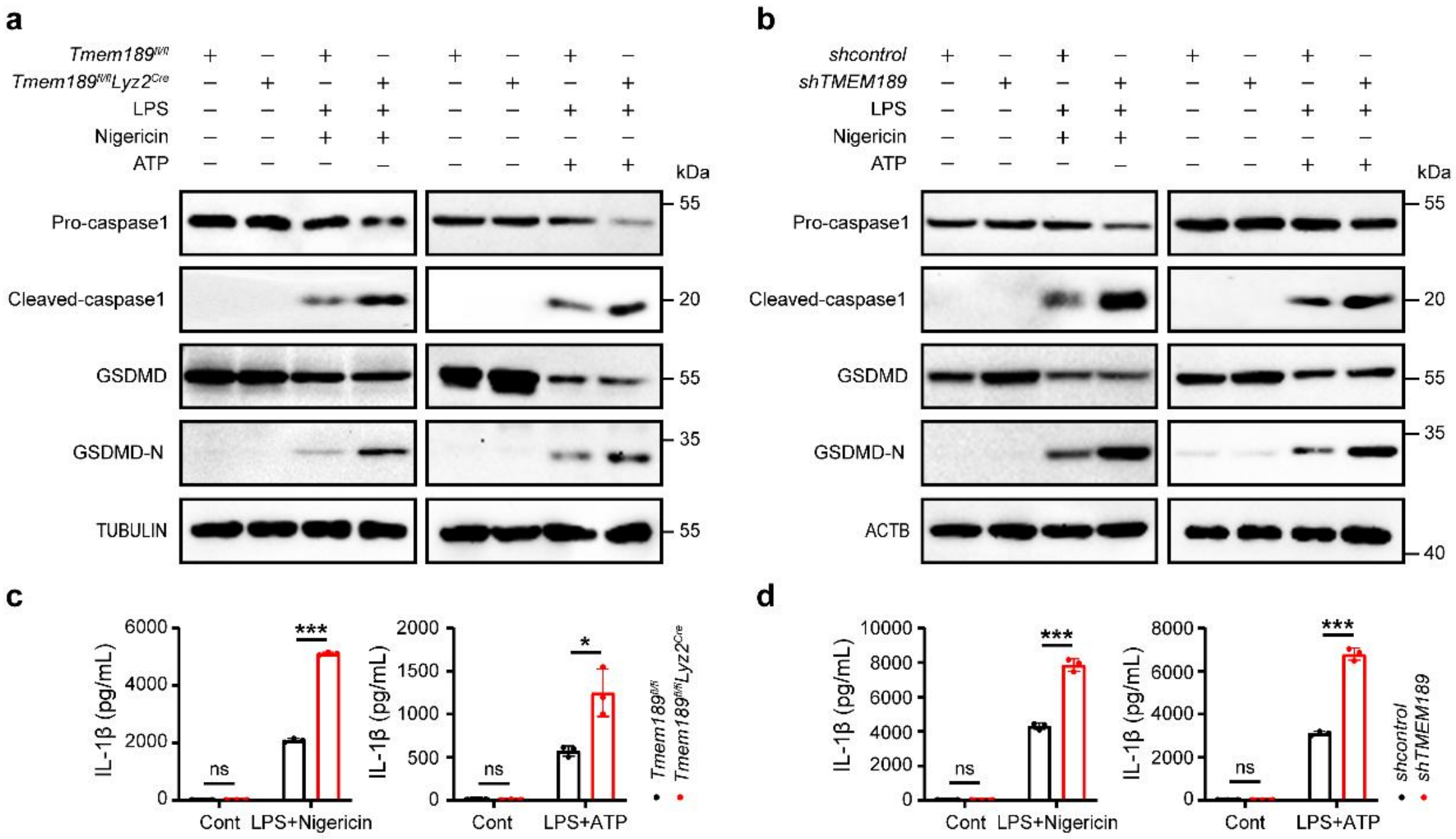

Fig. S14 Deletion of *TMEM189* enhances the activation of the NLRP3 inflammasome in macrophages. (a and **b**) BMDMs from *Tmem189*$^{fl/fl}$ and *Tmem189*$^{fl/fl}$*Lyz2*^{Cre} mice (a) or PMA-differentiated *shcontrol* and *shTMEM189* THP-1 cells (b) were treated with LPS (1 μg/mL) for 3 hours, followed by stimulation with nigericin (10 μmol/L) for 30 minutes or ATP (3 mmol/L) for 60 minutes. The levels of the indicated proteins were detected by Western blotting. (**c** and **d**) Cells were treated as described in (a and b), and the production of IL-1β in the culture supernatants was measured by ELISA. Statistical significance was determined using the multiple *t*-test with the two-stage linear step-up procedure of Benjamini, Krieger and Yekutieli (c, d). All plots represent the mean ± SD from at least three independent experiments. ns, not significant, *$p < 0.05$, ***$p < 0.001$.
