# Supplementary material for: TMEM189/PEDS1 deficiency aggravates MASH progression by promoting FASN-mediated macrophage activation
Source: Cell Death Dis. 2026 Aug 13;17(1):956. doi: 10.1038/s41419-026-09145-7 (PMC13748651; doi:10.1038/s41419-026-09145-7)
Supplement: Supplementary file 2 [file 41419_2026_9145_MOESM2_ESM.docx]

**Table S1. The list of antibodies in this study**

| **Antibodies** | **Supplier** | **Cat no.** |
| --- | --- | --- |
| FITC anti-mouse CD45 | Biolegend | 103108 |
| APC anti-mouse CD45 | Biolegend | 103111 |
| Brilliant Violet 510™ anti-mouse CD45 | Biolegend | 157219 |
| PE anti-mouse F4/80 | Biolegend | 123109 |
| APC anti-mouse F4/80 | Biolegend | 123115 |
| PE/Cyanine7 anti-mouse Ly-6C | Biolegend | 128018 |
| FITC anti-mouse Ly-6C | Biolegend | 128005 |
| PerCP anti-mouse/human CD11b | Biolegend | 101229 |
| APC/Cyanine7 anti-mouse/human CD11b | Biolegend | 101226 |
| Brilliant Violet 605™ anti-mouse  Ly-6G | Biolegend | 127639 |
| APC anti-mouse Ly-6G | Biolegend | 127613 |
| PE/Cyanine7 anti-mouse Tim-4 | Biolegend | 130009 |
| FITC anti-mouse CD4 | Biolegend | 130308 |
| PE anti-mouse CD8a | Biolegend | 162303 |
| FITC anti-mouse CD19 | Biolegend | 152404 |
| APC anti-mouse CD80 | Biolegend | 104713 |
| Alexa Fluor® 700 anti-mouse CD86 | Biolegend | 105023 |
| Anti-mouse F4/80 | Servicebio | GB113373 |
| Anti-TMEM189 | Sigma | T8953 |
| Goat Anti-Mouse IgG H&L (HRP) | abcam | ab205719 |
| Goat Anti-Rabbit IgG H&L (HRP) | abcam | ab6721 |
| Alexa Fluor 594 goat anti-rabbit IgG | ZSGB-Bio | ZF-0516 |
| Anti-iNOS | Cell Signaling Technology | 68186 |
| Anti-FLAG | Cell Signaling Technology | 14793 |
| Anti-GFP | Cell Signaling Technology | #2956 |
| Anti-TUBULIN | Cell Signaling Technology | 86298 |
| Anti-Fatty Acid Synthase (FASN) | Cell Signaling Technology | 3180 |
| Anti-β-Actin | Cell Signaling Technology | 4970 |
| Anti-mouse caspase1 | Cell Signaling Technology | 24232 |
| Anti-mouse cleaved caspase1 | Cell Signaling Technology | 89332 |
| Anti-Caspase1 | Cell Signaling Technology | 83383 |
| Anti-human Caspase1 | Cell Signaling Technology | 3866 |
| Anti-human cleaved caspase1 | Cell Signaling Technology | 4199 |
| Anti-human cleaved caspase1 | AdipoGen | AG-20B-0048-C100 |
| Anti-GSDMD | Cell Signaling Technology | 97558 |
| Anti-cleaved GSDMD | Cell Signaling Technology | 36425 |
| Anti-human GSDMD | Cell Signaling Technology | 69469 |
| Anti-GSDMD | Cell Signaling Technology | 39754 |
| Anti-NLRP3 | AdipoGen | AG-20B-0014-C100 |
