# Supplementary material for: TMEM189/PEDS1 deficiency aggravates MASH progression by promoting FASN-mediated macrophage activation
Source: Cell Death Dis. 2026 Aug 13;17(1):956. doi: 10.1038/s41419-026-09145-7 (PMC13748651; doi:10.1038/s41419-026-09145-7)
Supplement: Supplementary file 3 [file 41419_2026_9145_MOESM3_ESM.docx]

**Table S2. Reagents and Commercial kits used in this paper**

| **Reagents and Kits** | **Supplier** | **Cat no.** |
| --- | --- | --- |
| High-fat diet (HFD) | Research Diets | D12492 |
| methionine-choline deficient (MCD) | Research Diets | A02082002B |
| Streptozocin | Sigma-Aldrich | S0130 |
| Alanine Aminotransferase (ALT) Assay Kit | Applygen | E2021 |
| Aspartate Aminotransferase (AST) Assay Kit | Applygen | E2023 |
| Triglyceride (TG) Content Assay Kit | Applygen | E1013 |
| Total cholesterol (TC) Content Assay Kit | Applygen | E1015 |
| Oil red O staining kit | Solarbio | G1262 |
| Sirius Red staining kit | Solarbio | G1472 |
| Recombinant Murine M-CSF | ThermoFisher Scientific | 315-02 |
| Recombinant Murine IFN-γ | PeproTech | 315-05-100UG |
| Hoechst33342 | Invitrogen | H1399 |
| BCA protein assay kit | Beyotime | P0010 |
| Protease inhibitors | Roche Diagnostics | 04693116001 |
| Nitrocellulose membranes | Millipore | HATF00010 |
| TRIzol | Invitrogen | 15596018 |
| RevertAid First Strand cDNA Synthesis Kit | ThermoFisher Scientific | K1622 |
| SYBR Green qPCR Mix | Vazyme | Q712-03 |
| NEOFECTTM DNA transfection reagent | Bioss Inc. Neofect (beijing) biotech | TF20121201 |
| Protein G Sepharose™ Fast Flow | FreeMoreBio | 17061801 |
| Anti-FLAG Nanoselector Agarose | Dakewe Biotech | 016-101-002 |
| Anti-GFP Affinity Beads 4FF | Smart Lifesciences | SA070001 |
| Glutathione-Sepharose 4B beads | FreeMoreBio | FA13085 |
| Human IL-1β ELISA Kit | Proteintech | KE00021 |
| Human IL-6 ELISA Kit | Proteintech | KE00385 |
| Human TNF-α ELISA Kit | Proteintech | KE00367 |
| Mouse TNF-α ELISA Kit | Proteintech | KE10002 |
| Mouse IL-6 ELISA Kit | Proteintech | KE10007 |
| Mouse MCP-1 ELISA Kit | Proteintech | KE10123 |
| Mouse IL-1β ELISA Kit | Proteintech | KE10003 |
| Nigericin | Sigma-Aldrich | N7143 |
| ATP | Sigma-Aldrich | A6419 |
| LPS | Sigma-Aldrich | L2630 |
| Palmitic acid (PA) | Sigma-Aldrich | P0500 |
| Oleic acid (OA) | Sigma-Aldrich | O1008 |
| C75 | Selleck | S9819 |
| 2-BP | Sigma-Aldrich | 21604 |
| IP-ABE Palmitoylation Kit | Aimsmass | AM10314 |
