# Supplementary material for: TMEM189/PEDS1 deficiency aggravates MASH progression by promoting FASN-mediated macrophage activation
Source: Cell Death Dis. 2026 Aug 13;17(1):956. doi: 10.1038/s41419-026-09145-7 (PMC13748651; doi:10.1038/s41419-026-09145-7)
Supplement: Supplementary file 4 [file 41419_2026_9145_MOESM4_ESM.docx]

**Table S3 Primer sequences of mouse and human of PCR or qRT-PCR**

| Gene | Forward (5’-3’) | Reverse (5’-3’) |
| --- | --- | --- |
| *Tmem189^fl/fl^* | AAACTCCTAGCACCTCATCCCATT | AGGCTCTCAATTCATGCTGCTAAG |
| *Lyz2^Cre^* | CCCAGAAATGCCAGATTACG | CTTGGGCTGCCAGAATTTCTC |
|  | TTACAGTCGGCCAGGCTGAC | CTTGGGCTGCCAGAATTTCTC |
| *Clec4f^Cre^* | ATCTATGGTGCCAAGGATGACTCT | AAGTGGACCTGTAGTGACAGAAAG |
|  | CAGCCTGACAACTGGAGACATAG | AAGTGGACCTGTAGTGACAGAAAG |
| Mouse qRT-PCR | | |
| *Tmem189* | GGGTGCTGATACATGGGGTTC | GAAGCATTCCCAAGGGTACAG |
| *Tnfα* | CCCTCACACTCAGATCATCTTCT | GCTACGACGTGGGCTACAG |
| *Il6* | TAGTCCTTCCTACCCCAATTTCC | TTGGTCCTTAGCCACTCCTTC |
| *Il1β* | GCAACTGTTCCTGAACTCAACT | ATCTTTTGGGGTCCGTCAACT |
| *Ccl2* | TTAAAAACCTGGATCGGAACCAA | GCATTAGCTTCAGATTTACGGGT |
| *Tgfβ* | CTCCCGTGGCTTCTAGTGC | GCCTTAGTTTGGACAGGATCTG |
| *Col1a1* | GCTCCTCTTAGGGGCCACT | CCACGTCTCACCATTGGGG |
| *Nos2* | GTTCTCAGCCCAACAATACAAGA | GTGGACGGGTCGATGTCAC |
| *Actb* | GGCTGTATTCCCCTCCATCG | CCAGTTGGTAACAATGCCATGT |
| Human qRT-PCR | | |
| *TMEM189* | GAGGACGAGGCGTCTTGTTG | CAGCACCCCAGTGTACCAG |
| *TNF-α* | GAGGCCAAGCCCTGGTATG | CGGGCCGATTGATCTCAGC |
| *IL-6* | CCTGAACCTTCCAAAGATGGC | TTCACCAGGCAAGTCTCCTCA |
| *IL-1β* | ATGATGGCTTATTACAGTGGCAA | GTCGGAGATTCGTAGCTGGA |
| *MCP-1* | CAGCCAGATGCAATCAATGCC | TGGAATCCTGAACCCACTTCT |
| *FASN* | AAGGACCTGTCTAGGTTTGATGC | TGGCTTCATAGGTGACTTCCA |
| *IL-1β* | ATGATGGCTTATTACAGTGGCAA | GTCGGAGATTCGTAGCTGGA |
| ACTB | CATGTACGTTGCTATCCAGGC | CTCCTTAATGTCACGCACGAT |
